# Sustainable β-cyclodextrin/polyethylenimine-encapsulated activated algae hydrogel beads for high-capacity Cd(ii) removal: adsorption performance, mechanism, thermodynamics, and Box–Behnken optimization

**DOI:** 10.1039/d6ra00411c

**Published:** 2026-04-07

**Authors:** Ahlem Guesmi, Naoufel Ben Hamadi, Wesam Abd El-Fattah, Mohamed G. El-Desouky, Ashraf A. El-Bindary

**Affiliations:** a Chemistry Department, College of Science, Imam Mohammad Ibn Saud Islamic University (IMSIU) Riyadh 11623 Saudi Arabia; b Egyptian Propylene and Polypropylene Company Port Said 42511 Egypt; c Chemistry Department, Faculty of Science, Damietta University Damietta 34517 Egypt abindary@du.edu.eg; d Health Sciences Research Center (HSRC), Deanship of Scientific Research, Imam Mohammad Ibn Saud Islamic University (IMSIU) Riyadh 13317 Saudi Arabia

## Abstract

This work details the formation of a novel bio-adsorbent from algae functionalized with glutamic acid after being activated with hyaluronic acid. The functionalized algae were encapsulated in β-cyclodextrin as well as polyethylenimine and crosslinked by epichlorohydrin to form FAACP hydrogel beads that have been used for the removal of cadmium(ii) ions from wastewater, proving their potential use in environmental remediation. Full characterization was done by analytical tools such as XRD, FTIR, XPS, BET, and SEM-EDX. The FAACP hydrogel system surface area of 128.734 m^2^ g^−1^ is quite high. This study examined the effects of temperature, starting concentration of Cd(ii) ions, pH, and quantity of FAACP on adsorption. The equilibrium followed the pseudo-second-order kinetic model and Langmuir isotherm adsorption isotherm; chemisorption was the predominant mechanism of adsorption that required an activation energy of 30.18 kJ mol^−1^. This indicated that the adsorption process occurs by an increase in temperature, which means it is endothermic and spontaneous in nature. The Box–Behnken design under the response surface methodology using Design-Expert software enhanced the adsorption efficiency to a maximum value under optimum conditions: 0.02 g of FAACP in 25 mL at pH 6 with an adsorption capacity value of 254.75 mg g^−1^ for Cd(ii) ion solutions. X-Ray diffraction studies verified the stability and efficacy of the adsorbent throughout the process, and stability testing verified constant impurity elimination after six cycles of adsorption and desorption while maintaining the original chemical structure unchanged.

## Abstract 2

This work details the formation of a novel bio-adsorbent from algae functionalized with glutamic acid after being activated with hyaluronic acid.

## Graphical abstract

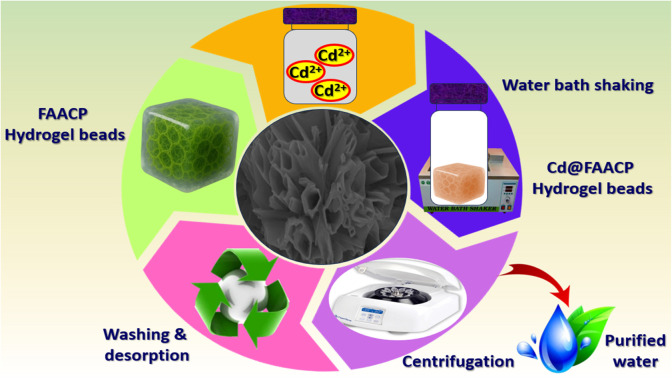

## 1. Introduction

Because heavy metals are poisonous and persistent in nature, they may pose a hazard to human health and the balance of the ecosystem, making their removal from wastewater essential. Metals like lead, cadmium, mercury, and arsenic have properties that allow them to bioaccumulate in aquatic organisms, alter habitats, and propagate through the food chain, thereby causing significant ecological damage.^1^ Untreated wastewater for agricultural practices may carry toxic elements into the soil, hence compromising crop safety. Heavy metals, even at low concentrations, can cause neurological dysfunction and renal failure in children, leading ultimately to cancer. Industrial wastewater compromises the safety of drinking water and hence increases health risks. Regulations have been set by countries on the concentration of heavy metals in effluents; thus, an efficient treatment process is needed to comply with such regulations. The removal of these metals makes it possible to recycle treated water for agricultural and industrial use and helps conserve this resource. Heavy metals corresponding lead, mercury, cadmium, chromium, and arsenic are highly dangerous since they are not biodegradable; hence they will remain in any ecosystem forever.^2^ Their emissions, which are free and without regulation, accumulate in organisms, disrupt the natural balance, and harm animal life.^3^ Drinking polluted water or eating tainted food can cause lifelong diseases; lead damages children's brain development, while mercury causes disablement. Cadmium is dangerous for the kidneys and bones, and arsenic can cause cancer.^4^ Even a small amount of exposure can lead to long-lasting, irreversible harm. As such, heavy metals found in wastewater pose a significant risk to public health and should be considered an environmental issue needing urgent resolution.^5^

Depending on the kind, concentration, and treatment objectives of the metal, different techniques are used to remove heavy metals from water solutions. One commonly used method is chemical precipitation. In this process, reagents like lime or sodium hydroxide are added to create insoluble metal precipitates that can be easily removed.^6^ This method is easy and inexpensive, but it produces sludge that must be disposed of. Another method is ion exchange, which uses resins to exchange heavy metal ions with non-toxic ions. This method is very effective for trace metals such as cadmium and nickel, although the resins are quite costly.^7^ Adsorption process involves activated carbon and agricultural by-products to adsorb heavy metals such as lead and mercury. This is most effective in low-contamination scenarios with an economic focus. Reverse osmosis as well as nanofiltration are two membrane filtration techniques that separate pollutants according to size and charge, however they need a lot of energy and upkeep.^8^ Electrochemical processes like electrocoagulation employ electric current for the precipitation of metal ions. However, these processes are highly energy-intensive. Bioremediation processes such as biosorption utilize biological agents like algae or bacteria to adsorb heavy metals, providing a greener and more sustainable alternative.^9^ Coagulation and flocculation are chemical methods that aggregate metallic particulates into larger clusters to facilitate sedimentation. They are often used as a preliminary treatment stage. Each technique has its own specific advantages and disadvantages, which usually necessitates the combination of these techniques with other methods to achieve the best possible outcome.^10^

Previously reported adsorbents based on β-cyclodextrin and polyethylenimine have demonstrated high efficiency in the removal of Cd(ii) and other metal ions from aqueous media. The synergistic combination of β-CD and PEI creates a multifunctional adsorption platform where β-CD enhances hydrophilicity and structural integrity *via* abundant hydroxyl groups, while PEI provides a high density of amine functionalities as strong chelation sites for divalent and trivalent metal ions. For example, crosslinked β-CD/PEI hydrogels prepared with epichlorohydrin have shown adsorption capacities for Cd(ii) in the range of ∼150–260 mg g^−1^ at optimized conditions.^11^ The Langmuir model best fit the adsorption isotherm data, and pseudo-second-order better explained the kinetics, indicating that chemisorption was the primary process. Similarly, due to improved dispersion and magnetic recoverability, higher removal efficiencies for Pb(ii), Cu(ii), and Ni(ii) have been reported in functionalized magnetic composites of β-CD/PEI with capabilities typically larger than 200 mg g^−1^.^12^ Although these performances seem promising, certain limitations still persist in the previously reported systems. Dense crosslinked polymeric networks may limit accessibility to internal amine sites and thus slow down the adsorption kinetics. Several β-CD/PEI hydrogels have low surface areas (<100 m^2^ g^−1^), which is not sufficient for effective mass transfer. Structural stability has been observed to decrease after a few regeneration cycles and performance further deteriorates in complex water matrices with competing ions. Therefore, it is rational to combine β-CD/PEI networks with a highly porous carbonaceous substrate so as to increase surface area, active site accessibility, mechanical stability, and general adsorption performance. In this study, the FAACP hydrogel system mixed activated algae with β-CD and PEI achieved a BET surface area of 128.734 m^2^ g^−1^ and maximum Langmuir uptake of 254.75 mg g^−1^ at 25 °C that increased up to 425.84 mg g^−1^ once the temperature was elevated to 45 °C. This is better than or at least similar to many other previously reported systems based on β-CD/PEI even though it retains structural stability after six regeneration cycles as proved by XRD analysis.^13^

The Box–Behnken Design used for optimizing the adsorption results presents a number of essential advantages. These benefits are particularly relevant to improving efficiency and accuracy in metal removal from effluent. As a type of response surface methodology, BBD facilitates systematic investigations into interactions between different experimental factors like pH, adsorbent quantity, interaction time, and original metal ion concentration without requiring exhaustive combinations of these variables. This significantly reduces the overall number of experiments needed and saves time, cost, and materials while still providing statistically valid results. One key advantage provided by Box–Behnken Design is its ability to develop models that predict optimal conditions for maximizing adsorption capacity.^14^ This approach facilitates the comparison of effects and interactions of individual factors on the adsorption process, which is often difficult to achieve with traditional one-factor-at-a-time approaches. Additionally, BBD minimizes the hazard of dangerous scenarios by without combinations where all factors are at their maximum or minimum levels simultaneously; therefore, it reduces the likelihood of impractical or unsafe conditions occurring. In conclusion, the application of BBD improves process efficiency, increases knowledge about system behavior, and supports the decision-making process necessary for scaling up adsorption systems in real-world wastewater dealing requests.

The originality of this study lies in the smart design and synthesis of a hierarchically structured, bio-based FAACP hydrogel, which integrates mesoporous activated algae carbon functionalized with glutamic acid, and β-CD/PEI crosslinked network into a single multifunctional adsorption platform. This method overcomes the limitations of conventional β-CD/PEI-based adsorbents, such as restricted surface accessibility, rigid polymeric frameworks, and moderate stability during regeneration. The present design employs a porous carbonaceous backbone to enhance active site exposure and facilitate mass transfer. The incorporation of glutamic acid further enriches the material with carboxyl functionalities that will synergistically interact with amine, and hydroxyl groups to create multiple cooperative coordination environments for efficient binding of Cd(ii). This study also improves upon previous studies by employing comprehensive physicochemical characterization, mechanistic investigation, and statistical optimization through response surface methodology to systematically evaluate adsorption performance and parameter interactions. Evidence of structural stability after several cycles of regeneration responds to another question related to polymer-based hydrogels suffering from durability problems. In total, this work transcends standard β-CD/PEI systems by developing an environmentally friendly multifunctional hybrid adsorbent that is structurally stable with enhanced accessibility and cooperative binding mechanisms for practical applications in heavy metal remediation.

## 2. Experimental

### 2.1. Materials and tools

As indicated in Table S1, analytical-grade reagents were utilized as such without additional purification. Instruments used in the study are described in Table S2.

### 2.2. Formation of an adsorbent

#### 2.2.1. Production of activated algae

The activated algae were prepared by washing the algae with hot water and drying them in a furnace at 100 °C. Then, 1.0 g of dried algae were mixed with 2 mL of 50% hyaluronic acid as chemical activator. The carbonization was enhanced by microwave irradiation at 800 W for 15 minutes under atmospheric inert nitrogen with a continuous current of 100 mL min^−1^. Following that, bi-distilled water was used to wash the active algae until the pH was neutral. Finally, the powdered activated algae were kept in a closed container for further use.^15,16^

#### 2.2.2. Functionalized activated algae (FAA)

In the procedure delineated, activated algae and glutamic acid were combined in equal proportions (1 : 1) with a solution comprising ethylene glycol and dimethylformamide, exposed to magnetic stirring for a period reaching from 1 to 5 hours at room temperature. Following this mixing phase, the resulting suspension was centrifuged to separate the NH_2_-activated algae, which are also denoted as functionalized activated algae. The isolated algae were dried in an oven at 80 °C overnight. In the next step, the mixture was heated inside a 100 mL Teflon autoclave by controlling its temperature. It was slowly raised to 100 °C with a rate of 5 °C per min and kept for 24 hours. After the heating process, it was washed three times with bi-distilled water and went through another round of centrifugation before being dried again overnight at 75 °C.^15,16^

#### 2.2.3. Synthesis of β-cyclodextrin/polyethylenimine cross-linked by epichlorohydrin

The preparation of β-cyclodextrin/polyethylenimine (β-CD/PEI) hydrogel begins with a stepwise procedure wherein 2.5 g (∼2.2 mmol) of β-CD is dissolved in 50 mL of bi-distilled water. This requires heating to about 50–60 °C with stirring until complete dissolution is achieved. At the same time, another solution containing around 5 wt% polyethylenimine is prepared by dissolving 2.0 g at room temperature using bi-distilled water. These two separate solutions are mixed after they are done. Next, 2 mL of epichlorohydrin (about 5–20 mol% based on the β-CD/PEI ratio) must be carefully added. The reaction takes place under energetic stirring between 50–70 °C. In this method, epichlorohydrin is used as a crosslinking agent to facilitate the creation of a steady hydrogel matrix. The pH of the reaction is controlled at 9–11 by the addition of 1.50 g (*ca.* 0.5 M) sodium hydroxide to improve the crosslinking reaction. After that, the mixture is stirred for 5–10 h to ensure complete development of the network structure. Following crosslinking, the resultant hydrogel is extensively cleaned with ethanol and deionized water to get rid of any unreacted epichlorohydrin before being dried at 50–70 °C in a vacuum oven.^17^

#### 2.2.4. Synthesis of functionalized activated algae encapsulated with β-cyclodextrin and polyethylenimine (FAACP) hydrogel beads

The first step requires that the functionalized (β-CD/PEI) hydrogel be soaked in bi-distilled water for two hours. This hydration procedure helps to swell the hydrogel, which is important in providing enough pore volume necessary for effective loading of functionalized activated algae (FAA). Afterward, 1.0 g of pre-made FAA powder is added into the swollen hydrogel matrix by ultrasonic agitation for 30 min to ensure that it is well distributed. This step is intended to promote an even distribution of FAA throughout the structural network of the hydrogel. After this initial incorporation, the resulting suspension will be subjected to stirring for periods ranging from 10 to 20 hours at ambient temperature, around 40 °C. Extended mixing times are crucial for establishing effective interactions between FAA particles and the hydrogel matrix. After the mixing, the composite is washed thoroughly with ethanol and bi-distilled water to remove any weakly bound amino acid particles that are not firmly attached. Finally, the functionalized activated algae inside the β-CD-PEI hydrogel beads are dried in a vacuum oven at a temperature of 60–70 °C for 10–20 hours. A stable and useful material that can be applied to many adsorption applications is produced by this procedure.^18^

### 2.3. Removal and batch analysis of Cd(ii) ions using FAACP hydrogel beads

**Fig. 1:**
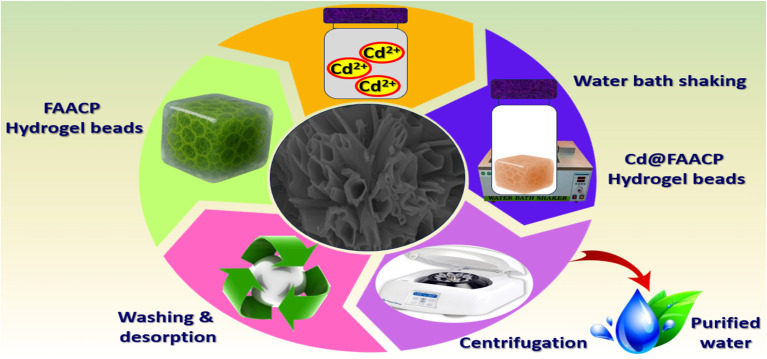
A schematic diagram representing the capture and mechanism of Cd(ii) ions removal by FAACP hydrogel beads.

The impacts of several parameters, including pH, dose of adsorbent, initial Cd(ii) ions concentration, equilibrium time, and temperature, were examined in order to assess the adsorption ability of FAACP beads.^19^ Cd(ii) ions were used in batch adsorption tests in a 25 mL aliquot made by diluting the cadmium ion stock solution. The primary goal was to investigate the adsorption isotherm at various concentrations ranging from 20 to 380 mg L^−1^. The runs were conducted under predefined conditions, with a constant volume of 25 mL, a temperature of 25 °C, and a contact time of 100 min. The pH was maintained at 6, and the adsorbent mass was 0.02 g. For studying the kinetics of adsorption, contact time parameters were set with a concentration held constant at 300 mg L^−1^. This was tested over a variety of interaction times from 5 to 100 min while keeping all other experimental conditions fixed at 25 °C, 25 mL, pH 6, and 0.02 g of adsorbent (Fig. 1). The effect of temperature was also studied by maintaining the concentration at 300 mg L^−1^ and fixing the contact time at 100 min 20. The temperature conditions for this study were set among 20 and 45 °C, with fixed parameters for volume, pH, and amount of adsorbent. Sodium hydroxide and hydrochloric acid (0.1 M) were employed to modify the pH during the experiment in order to maintain the required values.^21^ The same data were used to perform an isotherm and kinetic analysis to study the effect of temperature on the thermodynamic attributes related to the adsorption procedures (Table S3). The concentrations of metal ions in the solutions were measured before and after the adsorption process using a PerkinElmer flame atomic absorption spectrophotometer. The results are shown as mean (±) standard deviation, and each experiment was conducted in triplicate under identical conditions. The figures' error bars show the standard deviation of three separate measurements. The adsorption capacity (*q*_e_) and percentage of adsorbed metal ions by the FAACP were calculated using eqn (1) and (2).1
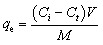
2
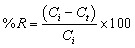


### 2.4. The design of experiments

Adsorbent dosage, pH, and contact time were the three independent variables in the adsorption experiment, which was conducted using the Response Surface Methodology and Box–Behnken Design. Stat-Ease Design Expert software version 13.0, produced in Minneapolis, USA, was used to build the Box–Behnken Design (Table S3). The coding and levels of the three factors are shown in Table S3. This design has *P* center runs, (2 × *m*) axial runs, and (2^*m*^) factorial runs to fully explore not only the interactions between these parameters but also the data generated from them. Eqn (3) could be used to express the overall number of experimental tests:

3 *N*_p_ = [2^*m*^ + (2 × *m*) + *P*] = [2^3^ + (2 × 3) + 3] = 17

In this study, (*P*) is the total number of runs required, and (*N*) is the number of process parameters that influence the results. Note that (*m*) was set to 3 for this investigation. The three primary steps of the central composite design are determining the model constants, creating the experiment, and evaluating the model's performance by examining the outcomes (Table S4). A quadratic equation, as indicated in eqn (4), was used to statistically analyze the elimination of Cd(ii) ions.

4 *Y* = *β*_0_ + ∑*β*_*i*_*X*_*i*_ + ∑*β*_*ii*_*X*_*i*_^2^ +∑∑*β*_*ij*_*X*_*i*_*X*_*j*_

The variable *Y* stands for the expected adsorption capacity (mg g^−1^) of Cd(ii) ions. The independent factors *X*_*i*_ and *X*_*j*_ have been encoded. The constant *β*_0_ and the coefficients *β*_*i*_, *β*_*ii*_, and *β*_*ij*_ for the linear, quadratic, and communication terms of the independent factors used as inputs are also defined. The results generated by the BBD model after completing 17 experimental runs are shown in Table 1.

**Table 1:** An in-depth study is crucial about the interactions at the surface associates and the adsorption of Cd(ii) ions inside the structural framework of the dominant compound

Run	Actual variables			*q*_e_ (mg g^−1^)		
	pH	Time (min.)	Dose (g)	Experimental	Predicted	Residue
1	2	5	0.26	19.902	8.95	10.95
2	5	52.5	0.26	165.129	165.13	0.0000
3	5	5	0.5	24.0154	37.49	−13.48
4	5	52.5	0.26	165.129	165.13	0.0000
5	5	5	0.02	38.424	47.32	−8.90
6	2	52.5	0.5	102.223	99.69	2.53
7	5	52.5	0.26	165.129	165.13	0.0000
8	8	100	0.26	174.483	185.43	−10.95
9	2	52.5	0.02	134.437	136.48	−2.05
10	5	52.5	0.26	165.129	165.13	0.0000
11	8	5	0.26	26.4998	15.07	11.43
12	8	52.5	0.5	112.983	110.94	2.05
13	2	100	0.26	131.088	142.52	−11.43
14	5	100	0.5	158.125	149.23	8.90
15	5	52.5	0.26	165.129	165.13	0.0000
16	8	52.5	0.02	171.734	174.27	−2.53
17	5	100	0.02	253	239.52	13.48

## 3. Results and discussion

### 3.1. Description of FAACP

#### 3.1.1. X-ray diffraction (XRD) pattern

**Fig. 2:**
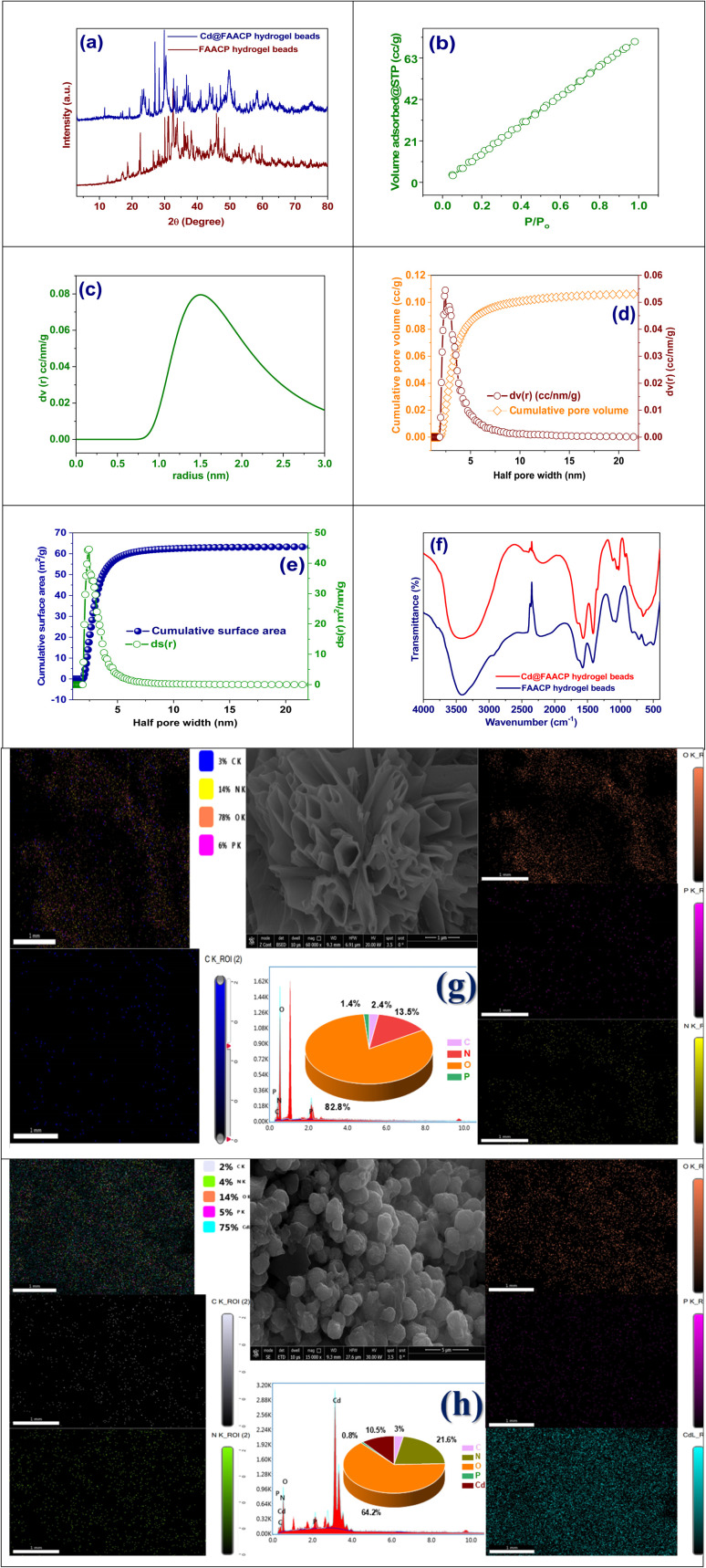
(a) XRD of FAACP and Cd@FAACP hydrogel beads, (b) N_2_ adsorption/desorption isotherm, (c) pore radius distribution, (d) cumulative pore volume, (e) cumulative surface area, (f) FT-IR of FAACP and Cd@FAACP, (g) SEM-EDX of FAACP hydrogel beads, and (h) SEM-EDX of Cd@FAACP.

The X-ray diffraction (XRD) pattern shown depicts the changes in the structure of FAACP hydrogel beads both prior to and following Cd(ii) ion adsorption. The brown curve for FAACP hydrogel beads has broad and low-intensity peaks that are characteristic of a mostly amorphous structure or one with some crystallinity. On the other hand, the blue curve for Cd@FAACP hydrogel beads has sharp and high-intensity peaks suggesting that new crystalline phases have formed or there is an increase in crystallinity of this hydrogel due to doping with Cd(ii). To analyze more details about its crystallographic structure, Foolproof software was used here for modeling based on a monoclinic crystal system defined by the space group *P*2. The unit cell parameters were refined to *a* = 10.9964 Å, *b* = 22.0169 Å, *c* = 10.4138 Å; angles *α* = 90.00°, *β* = 120.04° and *γ* = 90.00°. These results support the conclusion that it has a slightly distorted monoclinic structure, a feature commonly found in the frameworks of organic polymers.^10^ The use of refinement techniques such as Foolproof, Le Bail, or Rietveld allows for the clear confirmation of different structural modifications, which may be reflected by peak shifts indicating lattice strain or changes in ion coordination. Additionally, the emergence of new peaks could indicate phase transitions, as described in Table S5. X-Ray diffraction analysis provides compelling evidence that adsorption of Cd(ii) significantly modifies the structural arrangement of the hydrogel beads and improves their crystallinity, as illustrated in Fig. 2(a).

#### 3.1.2. The adsorption/desorption isotherm

Fig. 2(b) shows the nitrogen adsorption and desorption isotherm for FAACP hydrogel beads, providing significant insight into their porous structure and surface properties. The isotherm exhibits a gradual and nearly linear increase in the volume of gas adsorbed with an increase in relative pressure (*P*/*P*_0_). This is classified as a Type II isotherm according to IUPAC nomenclature. Type II isotherms are characteristically related with non-porous or macroporous supplies that allow both monolayer and multilayer adsorption on highly accessible surfaces, lacking any defined pore-filling threshold. Such behavior would suggest that the FAACP hydrogel beads might be characterized by macroporous or non-microporous morphology characterized by high accessible surface area rather than well-defined microporous structures. The BET surface area of FAACP was originate to be 128.734 m^2^ g^−1^. This value indicates a very high surface area, which is desired in adsorption requests. The assumption that hydrogel beads provide a large accessible surface area for the adsorption of heavy metal ions like Cd(ii) is supported by the observation of a high surface area and the categorization of the isotherm type. Such importance can be noted in its role to enhance material efficiency regarding adsorption and potential application in environmental cleanup operations.^22^Fig. 2(c) reveals a narrow and intense pore size distribution peak centered at approximately 1.5 nm, indicating that this pore size is predominant and falls within the mesoporous range (2–50 nm). Fig. 2(d) shows the analysis of pore size distribution (2.4435 nm) and cumulative pore volume. The pore size distribution curve (d*v*(*r*)) shows a significant increase in volume with decreasing pore diameter, confirming the mesoporous character of the material. The cumulative pore volume increases steadily and levels off at larger pore diameters, reaching a total cumulative pore volume of 0.106 cc g^−1^, indicating high availability of internal space in the hydrogel beads. In the graph seen in Fig. 2(e), there is a relationship between the total surface area and the area differential (d*s*(*r*)) with respect to half pore width. It can be noted that the total surface area increases sharply at small pore widths until it achieves its maximum value of 63.2623 m^2^ g^−1^. On the other hand, the differential surface area shows a maximum for small pore widths and then decreases, which corresponds to what was reported earlier about pore size distribution. The trends that have been seen here offer strong proof that smaller mesopores are mainly responsible for creating the surface area. The average particle radius measured is 10.593 nm; this supports that the material is indeed mesoporous. These results imply that FAACP hydrogel beads have a mesoporous structure with almost unvarying pore distribution, huge surface area, and adequate pore volume-these will make them very good at helping fast adsorption processes such as taking away heavy metal ions.^23^

#### 3.1.3. FT-IR analysis

The FT-IR spectrum in the figure exhibits the difference between FAACP hydrogel and the Cd@FAACP hydrogel. The functional groups that have changed are those after adsorption of Cd(ii). For FAACP beads, a broad peak around 3400 cm^−1^ resembles to O–H and N–H stretching vibrations, which is an indicative peak for hydroxyl and amine groups. After adsorption of Cd(ii), there is a noteworthy decrease in intensity of this peak suggesting that these functional groups may have participated in binding metal possibly *via* coordination mechanism. The spectral characteristics found in the region about 2920–2850 cm^−1^, which refer to C–H stretching vibrations of aliphatic chains, do not show much change indicating limited interaction. On the other hand, very sharp peaks about 1730 cm^−1^ belong to C=O stretching mode that gets stronger or moves its position after adsorption. This indicates the carboxylic useful groups are elaborate in chelation with Cd(ii) ions. Also, spectral regions roughly between 1620–1400 cm^−1^ corresponding to C–N stretching besides O–H bending vibrations plus those around 1040–1100 cm^−1^ related to C–O stretching or shifts proving coordination of cadmium ions with nitrogen, and oxygen, comprising useful groups. All these spectral changes indicate a strong communication among active useful groups on FAACP hydrogel like hydroxyl, amino, and carboxyl with Cd(ii) ions.^24^ In addition to improving the adsorption capacity for metals, it induces structural transformations within the hydrogel matrix, as demonstrated in Fig. 2(f).

#### 3.1.4. SEM and EDX analysis

Fig. 2(g) shows the scanning electron microscopy (SEM) investigation of FAACP at a high magnification. The surface morphology appears extremely porous and rough. The middle SEM image distinctly presents a network of sheet-like and interconnected structures, with clear voids and pathways indicating a highly developed mesoporous structure within the inside of the beads.^25^ Such a porous design greatly increases the surface area and suggestions many active spots for adsorption; therefore, these hydrogel beads are very good at capturing as well as interacting with heavy metal ions. In addition, the complicated structure plus the irregular shape indicates good diffusion paths in the hydrogel matrix which helps support effective mass transfer during adsorption.

Fig. 2(h) shows the SEM results of the Cd@FAACP beads after cadmium ions was adsorbed. The SEM image occupied at a magnification of 150 00× displays a very compact surface morphology made up of closely packed particles that are mostly spherical in shape. Compared to the surface of the initial FAACP hydrogel beads, these surfaces have more texture and roughness. This can be attributed to the successful adsorption and possible precipitation of cadmium ions onto the surface of the hydrogel. The change in morphology also means that the structure of the hydrogel physically reorganizes when it integrates Cd(ii) ions, which suggests strong interactions exist between these cadmium ions and the useful groups in the matrix of the hydrogel. Furthermore, this densely organized structure indicates less porosity since these cadmium ions now fill up all active sites as well as pores within the hydrogel.^26^

#### 3.1.5. EDX analysis

Fig. 2(g) displays the elemental mapping from EDX of FAACP hydrogel beads; this helps to appreciate the spatial distribution and number of important elements present in the hydrogel matrix. Results show uniform distributions of oxygen, nitrogen, phosphorus, and carbon across the sample. Moreover, the pie chart pertaining to this figure provides a quantitative representation of the elemental fractions whereby it can be seen that oxygen constitutes 82.8%, nitrogen 13.5%, phosphorus 1.4%, and carbon 2.4%. This data very clearly illustrates how much more dominant are oxygen and nitrogen in these FAACP hydrogel beads; hence they may play some role in their chemistry as well as in future applications. The abundance of both these elements also implies an increased density of polar useful groups like hydroxyl, amine, and phosphate groups which are known to bind metal ions quite efficiently. Such extensive chemical functionalities homogenously distributed on the surface enhance the adsorption capacity of beads toward heavy metals from environmental matrices.

The EDX analysis and elemental mapping results of the beads of the hydrogel Cd@FAACP are shown in Fig. 2(h). The attendance and distribution of carbon (C), nitrogen (N), oxygen (O), phosphorus (P), and cadmium (Cd) have been confirmed in this study. The maps generated for each element reveal that cadmium, which is represented here in cyan color, has an even distribution over the surface; this result confirms very efficient and uniform adsorption of cadmium ions onto the beads. The EDX spectrum with pie chart (*h*) shows quantification of elemental composition as follows: Cd (10.5%), O (64.2%), N (21.6%), P (0.8%), and C (3.0%). A large quantity of Cd here means that this kind of hydrogel has a great capacity for metal binding. The presence of these other elements also proves that functional groups –OH, –NH_2_, and –PO_4_ which are responsible for binding have been retained after adsorption occurred. Therefore, such distribution and profile strongly advocate the capability of FAACP beads to capture Cd(ii) ions confirming their potential as highly efficient materials in heavy metal remediation.

#### 3.1.6. XPS

**Fig. 3:**
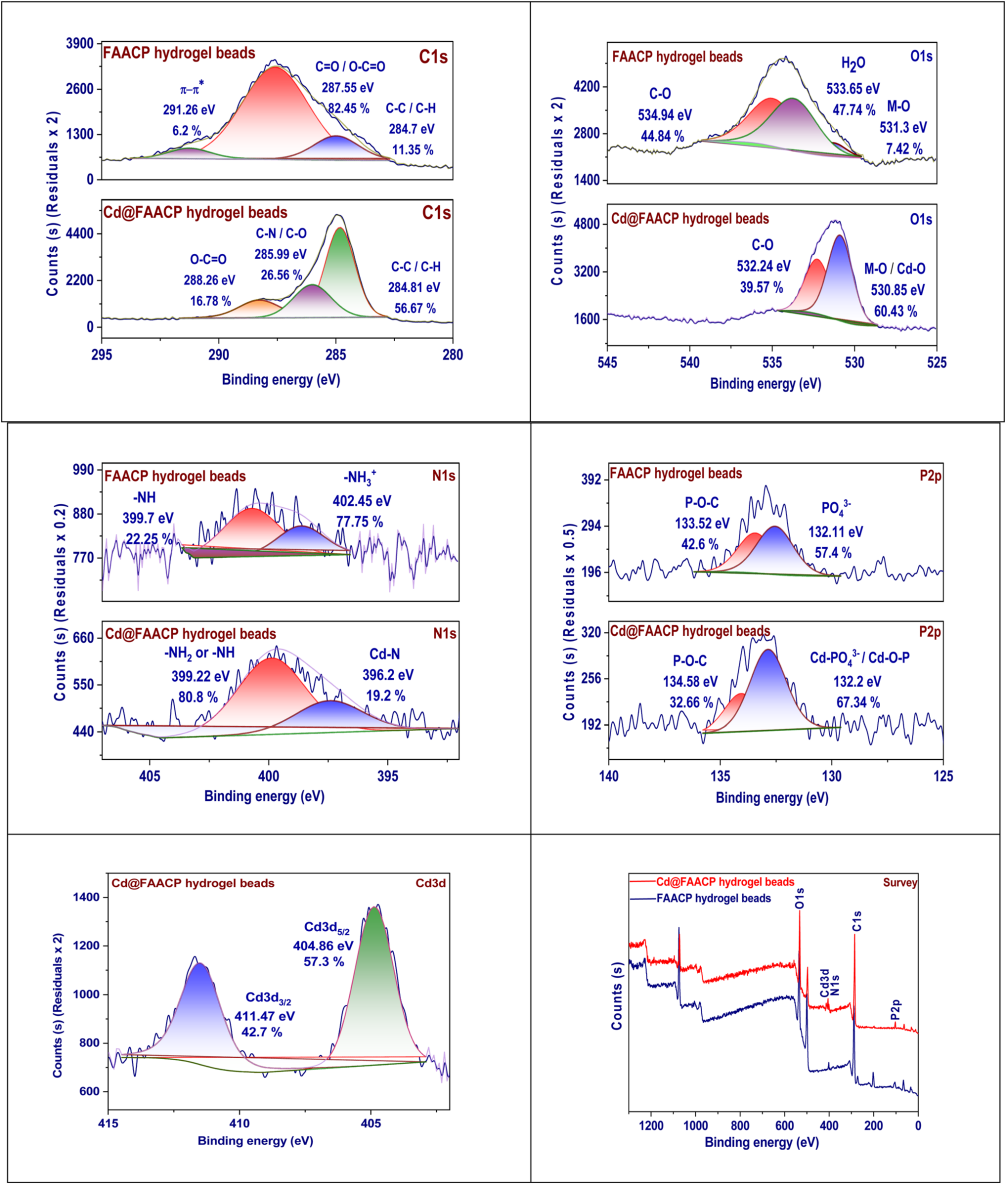
XPS of FAACP and Cd@FAACP.

The X-ray photoelectron spectroscopy (XPS) C1s spectra taken from the FAACP and modified adsorbent for cadmium ions, which we will call Cd@FAACP, show very big changes in chemical states and surface makeup after the adsorption of Cd(ii). When we look at the unmodified FAACP hydrogel beads, three main peaks are perceived at energy levels of 284.70 eV (related to C–C/C–H; this makes up 11.35%), 287.55 eV (indicating C=O/O–C=O with a share of 82.45%), and 291.26 eV (showing π–π* shake–up transitions that account for 6.2%). These peaks prove that there is some aliphatic carbon as well as a large amount of oxidized carbon species, which include carboxyl and carbonyl functional groups along with conjugated and aromatic structures. The inspection of the spectrum from Cd@FAACP after it has taken up Cd(ii) reveals large changes in both where some signals sit as well as how strong they are.^27^ New peaks appear at energies of 284.81 eV (C–C/C–H, now at a high intensity of 56.67%), 285.99 eV (C–N/C–O with a value of 26.56%), and finally one more at an energy level of 288.26 eV (O–C=O/amide with an intensity of 16.78%). This increase in the signal from aliphatic carbon plus the new peak for C–N/C–O shows that there has been a change in configuration and binding of Cd(ii) through amine as well as hydroxyl functional groups present in β-CD/PEI. The area related to the oxidized carbon peak significantly decreases while absence of π-π* satellite signal implies strong interaction among Cd(ii) and oxygen-comprising functional groups which may lead to conjugated structures disruption. The spectral changes indicate convincingly complexation success for coordination with nitrogen and oxygen donor atoms by Cd(ii)in FAACP beads highlighting active sites within hydrogel capable metal ion adsorption potential (Fig. 3).

The XPS O1s spectra for the FAACP hydrogel and the Cd(ii)-ion-adsorbed beads (denoted as Cd@FAACP) show notable changes in the distribution of oxygen-comprising practical groups after metal adsorption. The analysis of FAACP beads shows three different peaks at binding energies of 531.30 eV (7.42%), 533.65 eV (47.74%), and 534.94 eV (44.84%). These peaks indicate the existence of various practical groups: the first peak is assigned to metal–oxygen (M–O) or hydrogen-bonded hydroxyl (OH) groups, the second one is assigned to carbon-oxygen (C–O) functionalities which may include hydroxyl or ether groups; this third peak is related to adsorbed water plus carboxyl or ester functionalities. The observed peaks confirm that appreciable amounts of oxygen functionality exist and can be assigned to components from algae biomass, β-CD, and PEI. The O1s spectrum for the Cd@FAACP beads after adsorption of Cd(ii) shows two separate peaks located at 530.85 eV (60.43%) and 532.24 eV (39.57%).^28^ The peak with lower binding energy value indicates cadmium-oxygen (Cd–O) coordination bond formation while another is due to residual C–O or O–C=O functional groups; more importantly, there is no peak at 534.94 eV in combination with increased intensity at lower binding energies which emphasizes that it is mainly through hydroxyl and carboxyl functional group donor atoms that Cd(ii) ions are held by different mechanisms like complexation and electrostatic attraction, thus proving how effective FAACP hydrogels are for metal adsorption (Fig. 3).

XPS analysis of FAACP and its Cd(ii)-adsorbed version Cd@FAACP shows that metal binding brings about major changes in nitrogen functional groups. Spectral analysis reveals two peaks for FAACP: the first one at 399.7 eV with an intensity of 22.25% relates to nitrogen in the –NH– or amine state; the second peak occurs at 402.45 eV and accounts for 77.75% of intensity, indicating protonated amine or quaternary nitrogen forms are present. A notable peak at 402.45 eV reveals that the dominant source of positively charged nitrogen is PEI, which is responsible for cation exchange and electrostatic interactions. The spectral properties undergo significant changes upon adsorption of Cd(ii) on Cd@FAACP beads, as indicated in the lower part of the spectrum. The major peak at 399.22 eV constitutes 80.8% of what is displayed by the spectrum and confirms the presence of more neutral amine nitrogen functional groups (–NH_2_ or –NH–). A minor peak at 396.2 eV that accounts for 19.2% of the spectrum pertains to cadmium (Cd) bonding with nitrogen (Cd–N) and denotes some complexation between adsorbed species and the material composition of beads. The observed spectral shift, along with a newly formed peak associated with Cd–N interactions, suggests that nitrogen atoms in PEI are directly involved in complexing with Cd(ii). Such a mechanism could be driven by lone pair donation from nitrogen atoms resulting in reduced abundance of protonated nitrogen species; these changes highlight the role of nitrogen functionalities within FAACP matrix for trapping Cd(ii) ions thereby substantially enhancing metal-binding capacity *via* coordination as well as electrostatic mechanisms in hydrogel.^29^

XPS of P2p spectra of the hydrogel beads, FAACP, and those hydrogel beads adsorbed with Cd(ii), referred to as Cd@FAACP, provide very important information on the different phosphorus species elaborate in the adsorption process. In the spectrum for FAACP hydrogel beads at the top, two strong peaks can be seen at binding energies of 132.11 eV (57.4% of the total) and 133.52 eV (42.6%); these peaks are associated with PO_4_^3−^ species which means phosphate groups and P–O–C or polyphosphate-like linkages, respectively. These species may originate from the natural phosphorus content of the algae material and possible contributions from phosphate modified additives. In the case of adsorption of Cd(ii), an examination of the P2p spectrum reveals that while peak positions do not change significantly, relative intensities vary greatly. The peak at binding energy 132.2 eV increases to 67.34%, while that at higher binding energy 134.58 eV decreases to 32.66%. This change in intensity distribution suggests more phosphate species with lower binding energy have become abundant after interaction with Cd(ii); possibly through some coordination complex formation such as Cd–PO_4_ or Cd–O–P.^30^ The decrease in intensity detected for the P–O–C component at high energy further supports this idea, as it indicates that phosphate groups are responsible for chelation or electrostatic binding of Cd(ii) ions (Fig. 3).

The XPS spectrum shown here is for the Cd3d region of the hydrogel beads known as Cd@FAACP. It strongly supports that cadmium ions have been successfully immobilized in a hydrogel matrix. Two major peaks can be seen at binding energies of 404.86 eV (57.3% of the total signal) and 411.47 eV (42.7%), which are assigned to the electronic states of Cd3d_5/2_ and Cd3d_3/2_, respectively. These peaks belong to typical spin–orbit splitting features for cadmium in its +2 oxidation state (Cd(ii)).^30^ The seen bonding energies agree with a coordination among Cd(ii) ions besides oxygen or nitrogen atoms in organic matrices. This means that the Cd^2+^ ions are not found as free or metallic forms but rather are chemically linked to functional groups in the FAACP matrix. The finding of these peaks supports strong stable coordination bonds among Cd(ii) ions and donor atoms like –COO^−^, –OH, or –NH– inside the hydrogel. The different relative intensities of these peaks, along with their sharp forms and certain binding energy positions, confirm that cadmium has been effectively taken up through chelation or electrostatic interactions. Such analysis brings out great prospects for this hydrogel to be used as an efficient biosorbent for heavy metal remediation from water environments.

The XPS survey spectrum of FAACP and its cadmium ions adsorbed variant (Cd@FAACP) shows major elemental compositions confirming the actual binding of Cd(ii). The spectra are represented with a blue curve for FAACP and a red curve for Cd@FAACP. New peaks are observed conforming to oxygen (O1s at about 532 eV), nitrogen (N1s at about 400 eV), carbon (C1s near 285 eV), and phosphorus (P2p around 133 eV). These are due to the biopolymeric matrix of hydrogel comprising algae, β-CD, and PEI. The analysis of the sample Cd@FAACP reveals an intense peak for Cd3d at about 405 eV which is not there in unmodified FAACP; this shows that cadmium has been successfully added to the material's structure. In addition, in the sample loaded with Cd, a large increase in relative intensities is noted for both O1s and N1s peaks. This means that useful groups containing nitrogen besides oxygen are important for coordinating Cd(ii), probably through chelation or electrostatic interactions. The constant presence of P2p in both samples shows that phosphate groups take part in binding the metal. The survey spectrum, therefore, reveals a complex elemental composition inside FAACP hydrogels, further proving their efficiency toward adsorption of Cd(ii) ions due to many functional groups present which act as active binding sites.^30^

#### 3.1.7. Point of zero charge

**Fig. 4:**
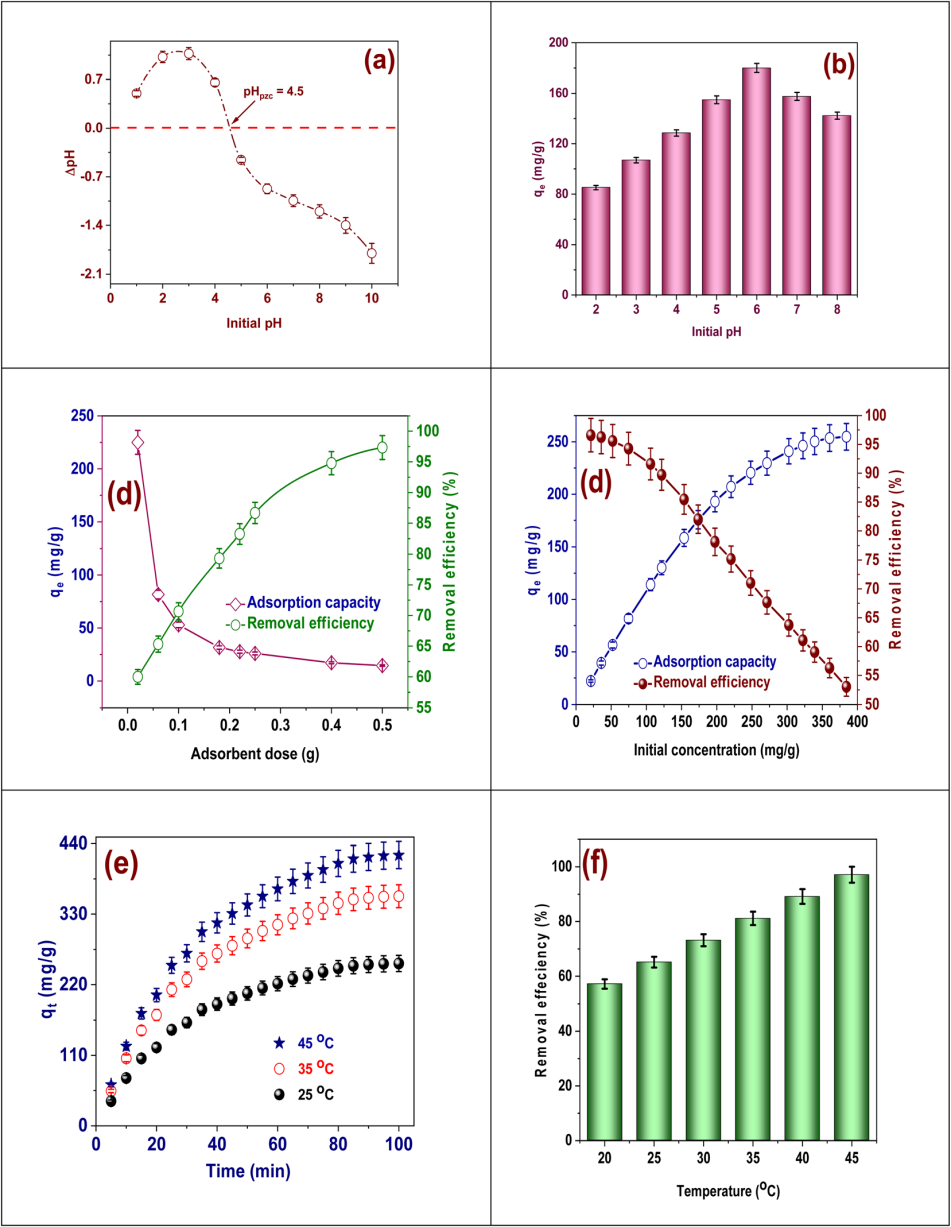
(a) Resolve of pH_pzc_, (b) the pH effect, (c) the adsorbent quantity effect, (d) the initial concentration effect, (e) the interaction time effect, and (f) the temperature effect.

The point of zero charge (pH_pzc_) in Fig. 4(a) for FAACP hydrogel beads is at 4.5, which means that when the surface of the adsorbent is electrically neutral it helps to know about the adsorption of Cd(ii) ions. When pH is less than 4.5, since the beads carry a positive charge there will be a repulsion force with the Cd(ii) ions leading to lower adsorption efficiency. When the pH is more than 4.5, since the surface charge becomes negative it enhances electrostatic interaction thus leading to significant removal of Cd(ii). The FAACP beads are made from algae-derived activated carbon coated with β-CD/PEI and have a porous structure and high surface area and have useful groups like hydroxyl, amine, and carboxyl which can bind metal ions by complexation as well as by ion exchange. The hydrogel matrix provides structural stability for reuse so that many cycles can be effectively adsorbed. FAACP hydrogel beads possess the tunable surface charge, functional groups, and green composition that make them efficient and sustainable adsorbents for the elimination of Cd(ii) from effluent.^31^

### 3.2. Batch experiments

#### 3.2.1. The pH effects

The adsorption capacity (*q*_e_) of Cd(ii) ions onto FAACP was first examined in relation to pH (Fig. 4(b)). The findings demonstrated that the adsorption capacity rose as pH increased from 2 to 6, reaching around 180 mg g^−1^ at pH 6, which is thought to be ideal for this reaction. Removal of Cd(ii) in the system under study occurs best at this particular pH value. This trend may be due to changes in both surface charge properties of the adsorbent and chemical speciation of cadmium ions present in solution.^32^ The ability to take in drops sharply between pH 2 and 4 because more hydrogen ions participate with cadmium ions for the active places on the surface of the hydrogel. In this pH range, there is a positive surface charge on the hydrogel that creates an electrostatic repulsion between substrate and cadmium ions.^33^ As the pH increases above 4.5, it causes the surface charge of the hydrogel to turn negative. This increases electrostatic attraction towards the positively charged ions of Cd(ii), thus improving adsorption efficiency and cadmium uptake. At pH 7 and 8, however, adsorption efficiency begins to decline after the pH exceeds 6 since at this stage precipitation of Cd(OH)_2_ starts which indicates that no more free Cd(ii) ions are left for adsorption. In broad terms, therefore, pH 6 can be taken as optimal for maximum cadmium adsorption because these coincide with mechanisms that involve electrostatic interactions and also pertain to the point of zero charge for FAACP beads.

#### 3.2.2. Dose effect

The impact of adsorbent amount on adsorption capacity (*q*_e_) and removal efficiency (%) of Cd(ii) ions using FAACP hydrogel beads (Fig. 4(c)). An inverse relationship between these two parameters is observed as the adsorbent dosage rises from 0.02 to 0.50 g. At a lower dosage of 0.02 g, the system exhibits a high value for adsorption capacity (∼225 mg g^−1^), though, with low removal efficiency due to rapidly saturated adsorption sites. Increasing the dosage enhances the number of active sites thereby improving removal efficiency which peaks at an impressive value of 957.33% with 0.5 g. Nevertheless, adsorption capacity per gram of adsorbent declines because excess active sites remain unutilized when Cd(ii) concentration is not sufficient to occupy them all; this results in dilution effects caused by higher quantities of adsorbent dispersing a fixed concentration of Cd(ii), consequently reducing metal uptake per gram of adsorbent. Even though higher doses appear to improve removal rates, it is essential to find the best dose that strikes a good balance between high adsorption capacities and successful pollutant removals. This also implies that FAACP hydrogel beads are effective at lower doses, making them a practical option for affordable cadmium extraction in wastewater treatment.^34^

#### 3.2.3. Initial concentration effect

Fig. 4(d) displays the result of varying initial Cd(ii) ions concentration on both the adsorption capacity (*q*_e_) and removal efficiency of FAACP beads. A distinct trend emerges as the initial Cd(ii) concentration is varied from 0 to 400 mg L^−1^, with a gradual increase in adsorption capacity and an opposite trend observed for removal efficiency. At lower concentrations (0–100 mg L^−1^), the adsorbent surface is likely to have abundant easily accessible active sites, allowing it to adsorb a significant fraction of Cd(ii) ions, leading to high removal efficiencies (approximately 95–98%) and moderate *q*_e_ values. Conversely, as concentrations increase (100–400 mg L^−1^), the attendance of more Cd(ii) ions in solution improves the uptake per unit mass of adsorbent, causing *q*_e_ to rise steadily, reaching values beyond 250 mg g^−1^. The practical decrease in overall removal efficiency to about 52% at 400 mg L^−1^ can be credited to saturation of adsorption places on the adsorbent; once the concentration exceeds its binding capacity for these ions, any excess remains unadsorbed in solution. This indicates that while higher concentrations enhance driving forces for mass transfer and increase loading capacities, they do not proportionately improve total percentage removals of Cd(ii). Such analyses emphasize optimizing initial concentrations based upon treatment objectives. These results further substantiate the broad applicability potential versatility FAACP beads as effective adsorbents over wide ranges applicable real-world scenarios.^35^

#### 3.2.4. Interaction time effect

Fig. 4(e) shows how contact duration affects Cd(ii) ion adsorption onto FAACP beads at various temperatures (25, 35, and 45 °C). The image makes it clear that the presence of active binding sites on the adsorbent's surface causes the adsorption capacity (*q*_e_) to rise dramatically over the first 40 min. As time proceeds, more binding sites are occupied and intraparticle diffusion begins to dominate the process, thus slowing down the rate until equilibrium is reached.^36^ The capacity of adsorption rises considerably with temperature, representative that the adsorption procedure is inherently endothermic. At 45 °C, maximum capacity of adsorption exceeds 400 mg g^−1^ in 100 min; at 35 °C, it is about 330 mg g^−1^ and at 25 °C only about 250 mg g^−1^. Higher temperatures enhance the mobility of Cd(ii) ions so that they can easily diffuse into the hydrogel matrix and may also activate more functional groups for better binding efficiency. Both longer contact time and higher temperature favor the adsorption efficiency of FAACP beads, proving their high performance and suitability for application in thermal control or industrial wastewater treatment systems.

#### 3.2.5. Temperature effect

The impact of temperature on the effectiveness of Cd(ii) removal using FAACP beads is seen in Fig. 4(f). The data indicate a very strong positive relationship between temperature and adsorption performance. It is noted that the removal efficiency increases from about 58% at 20 °C to above 95% at 45 °C, which means that adsorption is an inherently endothermic process. There are several reasons why better performance is observed at higher temperatures: first, more kinetic energy among Cd(ii) ions results in increased mobility and diffusion toward the surface of adsorbent; second, swelling properties of hydrogel matrix may become more pronounced facilitating access to internal active sites; finally thermal activation may promote more effective interaction between functional groups (amine and hydroxyl groups) in FAACP beads with Cd(ii) ions. Elevated temperatures appear to reduce the energy barriers for metal ion binding thus improving uptake. This evidence further supports that higher temperatures not only accelerate kinetics but also improve overall capacity for cadmium removal. Therefore, FAACP beads can be considered as a potential candidate adsorbent material for wastewater treatment applications under thermally optimized conditions.^37^

### 3.3. Adsorption isotherm

**Fig. 5:**
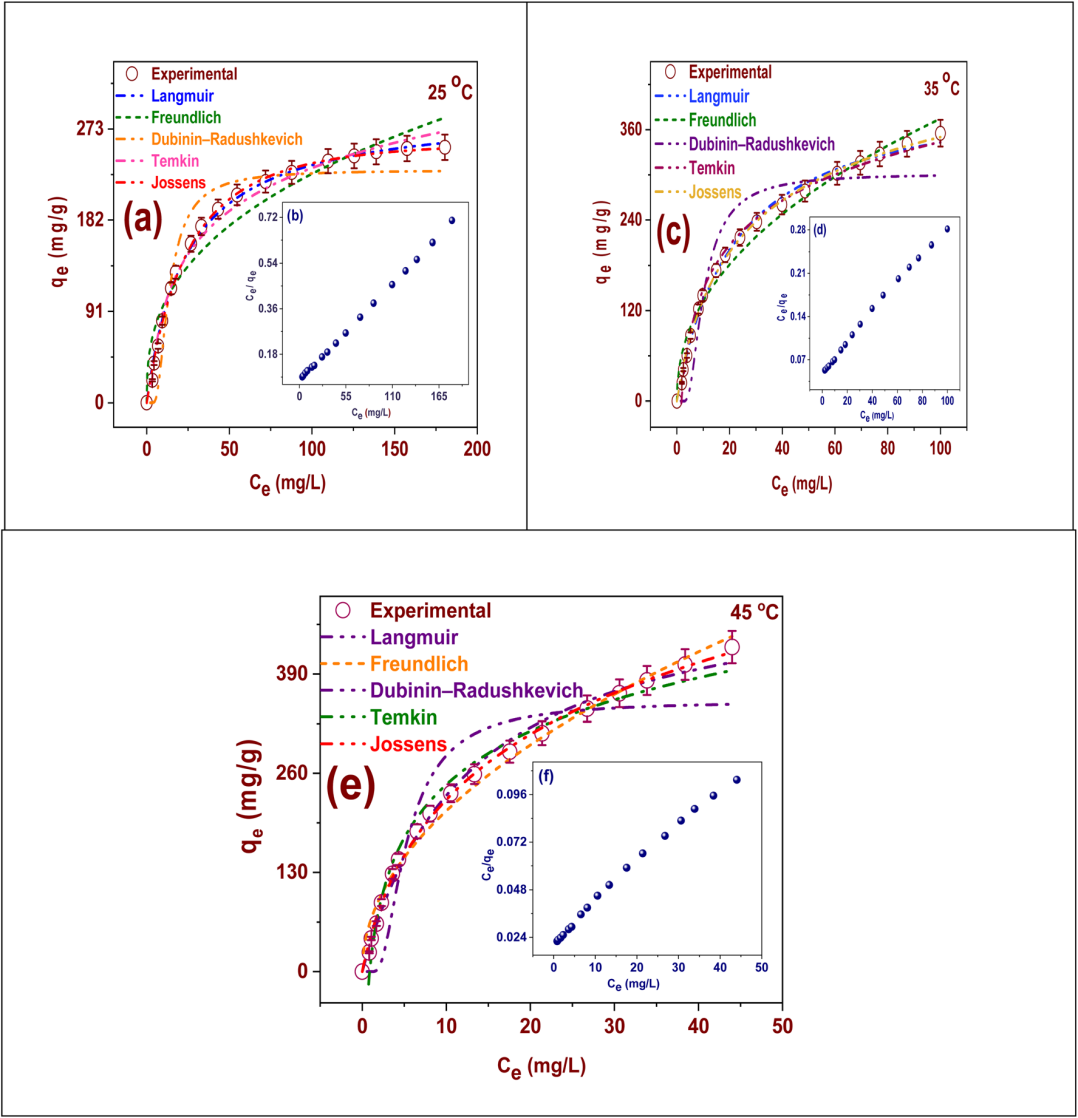
The models of adsorption isotherm of Cd(ii) ions adsorption onto FAACP at different temperature linear regression: (a) at 25 °C, (c) at 35 °C, (e) at 45 °C; and nonlinear regression (b) at 25 °C, (d) at 35 °C, and (f) at 45 °C.

Models of adsorption isotherm are very important for understanding the mechanism, capacity, and surface properties of hydrogel for the elimination of Cd(ii) by FAACP. These models help predict the equilibrium interactions between Cd(ii) ions and adsorbent, which is an significant consideration for efficient design and improvement of water treatment systems as shown in Fig. 5(a–c). Langmuir isotherm proposes that adsorption happens as a monolayer on a homogeneous surface; thus, it allows one to determine a measurable maximum adsorption capacity.^38^ This feature makes it particularly suitable for companies that possess stable energy sites. Conversely, the Freundlich isotherm includes the concept of multilayer adsorption onto surfaces that display heterogeneity or unevenness.^39^ Lower concentrations have been found to be very effective in revealing the surface heterogeneity of FAACP beads. In this regard, the Dubinin–Radushkevich (D–R) isotherm is crucial because it uses mean free energy calculations to distinguish between chemical as well as physical adsorption, which are essential for precisely determining the properties of an adsorption method.^40^ The Temkin isotherm considers the connections among the adsorbent besides adsorbate. It assumes that the heat of adsorption diminishes linearly with adsorbate attention.^41^ The proposed hypothesis is a more explicit representation of the adsorption phenomena, whereas the Jossens isotherm is highly suitable for surfaces characterized by substantial heterogeneity, offering a more comprehensive understanding of variations in adsorption energies (Table S6). The combined application of both models facilitates an exhaustive and precise characterization of the adsorption mechanism, thereby simplifying the process of scaling up and implementing cost-operative strategies for the elimination of heavy metals using FAACP hydrogel beads. The adsorption isotherm and parameters of kinetic were obtained through nonlinear regression analysis to avoid errors that might arise from the linear transformations of the models. All curve fitting and parameter estimates were accomplished using Origin Pro software (Origin Lab Corporation, USA).

The Langmuir isotherm parameters for the Cd(ii) adsorption onto FAACP beads at 25, 35, and 45 °C are presented in Table S7. These parameters can provide an understanding of the mechanism of adsorption and its temperature dependency. The maximum capacity of adsorption (*q*_max_) increased significantly with temperature from 254.75 mg g^−1^ (25 °C) to 355.26 mg g^−1^ (35 °C) and then to 425.84 mg g^−1^ (45 °C).^38^ The process of adsorption is endothermic and gets better with rise in temperature, according to the study outcomes. This trend suggests that higher thermal energy helps Cd(ii) ions move around and interact more easily with the active sites in FAACP hydrogel. A close look at the constant of Langmuir (*K*_L_), which is a measure of the contact strength between adsorbent and adsorbate, increases from 0.042 to 0.079 L mg^−1^ with rising temperatures. This means that as temperature increases, binding affinity also increases. Moreover, the dimensions-free separation factor *R*_L_ which designates how promising an adsorption procedure is (values between 0 and 1 imply favorable adsorption) decreases from 0.17 (25 °C) to 0.10 (45 °C) further supporting that the adsorption process becomes more favorable with increasing temperatures (Fig. 5(a, c and e)). Data regarding Langmuir isotherm reveal that FAACP beads have high efficiency for removing Cd(ii) ions particularly at elevated temperatures. The Langmuir model was fitted to the adsorption isotherm data at three distinct temperatures: 25, 35, and 45 °C. The adsorption process occurs in a Langmuir-type monolayer adsorption behavior, as confirmed by the fitting findings' extremely strong agreement with experimental data at all temperatures examined (Fig. 5(b, d and f)).

The Freundlich isotherm parameters for the Cd(ii) adsorption onto FAACP beads at 25, 35, and 45 °C are shown in Table S7. This information indicates that the system's surface is heterogeneous and that multilayer adsorption may take place. The Freundlich constant (*K*_F_) shows an increasing trend with temperature. At 25 °C, it has a value of 41.089 (mg g^−1^) (L mg^−1^)^1/*n*^ and at 35 °C, it increases to 46.9. Additional rise in temperature to 45 °C raises the value of *K*_F_ to 67.21. Such behavior implies that with rising temperatures, the capacity for adsorption by the hydrogel grows stronger; this is indicative of an endothermic adsorption process.^39^ The intensity parameter (*n*), a measure of the favorability of the adsorption method, drops sharply from 2.68 (25 °C) to 2.21 (35 °C) and then falls substantially to 0.496 (45 °C). Generally, *n* values above one is considered indicative of favorable adsorption conditions while those below one suggest either cooperative adsorption phenomena or less favorable behaviors. Hence, although the total hydrogel adsorption capacity appears to increase with temperature, the declining *n* value suggests an evolution toward less favorable or more heterogeneous adsorption characteristics as temperature increases. This might possibly indicate a change in mechanism or saturation at high-energy binding sites. In conclusion, based on the Freundlich model, it supports that the FAACP hydrogel has a high capacity for Cd(ii) even though the nature and uniformity of this uptake may vary with changes in temperature.

The D-R isotherm the model's settings for Cd(ii) adsorption onto FAACP hydrogel beads at 25, 35, and 45 °C are shown in Table S7. These parameters are essential for elucidating the thermodynamic factors and mechanisms controlling the adsorption method.^40^ The theoretical capacity of adsorption increased with the temperature from 232.04 mg g^−1^ (25 °C) to 301.62 mg g^−1^ (35 °C) and further increased up to 354.2 mg g^−1^ (45 °C), which indicates that the procedure is endothermic in nature and develops more promising with rising temperatures. The D–R constant has an inverse relationship with temperature; however, the mean adsorption energy shows only a slight increase in value from 30.18 to 33.2 kJ mol^−1^ over the same temperature range. Since all calculated values of *E*_a_ exceed significantly the limit of 8 kJ mol^−1^, it would be classified as chemisorption rather than physisorption, implying strong chemical interactions rather than weak physical forces. Therefore, one can conclude that Cd(ii) ions are probably forming chemical bonds with active sites on FAACP hydrogel surface to increase stability and effectiveness of adsorption system. Results from D–R isotherm analysis indicate that Cd(ii) adsorption on FAACP hydrogel is mainly measured by chemical interaction intensified by increasing temperatures which supports the concept of using hydrogel as a good medium for heavy metal contamination remediation.

The parameters of the Temkin isotherm for the Cd(ii) adsorption onto FAACP at 25, 35, and 45 °C are given in Table S7. These data help to analyze thoroughly the energy interactions between adsorbate Cd(ii) and adsorbent FAACP hydrogel beads. According to the Temkin model, it is expected that the adsorption heat will decrease linearly with increasing coverage on the surface reflecting secondary interactions between adsorbate molecules. The parameter *b*_T_ (in J mol^−1^), equivalent to the heat of adsorption, is observed decreasing from 39.9 J mol^−1^ (25 °C) to 29.15 J mol^−1^ (35 °C) and then further down to 24.54 J mol^−1^ (45 °C). This trend indicates an exothermic adsorption process that becomes weaker with rising temperatures.^41^ This shows that the more coverage there is on the FAACP hydrogel surface, the less energetic interaction there seems to be between Cd(ii) and the surface. This might mean that either the binding strength has gone down or it could simply be an effect of saturation. However, the Temkin equilibrium binding constant (*K*_T_) rises with temperature from 0.432 to 1.14 L mol^−1^ which means a greater capacity for adsorption and a stronger affinity at higher temperatures. From cumulative results taken from the Temkin model, even though energy related to adsorption diminishes as temperature increases, overall affinity and efficiency of Cd(ii) adsorption increases further supporting that this particular process is thermally favorable thereby increasing the applicability of FAACP hydrogel for metal ion removal under varying thermal conditions.

The Jossens isotherm parameters at various temperatures for Cd(ii) adsorption on FAACP are presented in Table S7. It helps to know about the distribution of energy and the nature of heterogeneity in adsorption places. The K parameter which indicates affinity as well as capacity for adsorption increases significantly with temperature from 10.78 (25 °C) to 23.06 (35 °C) and then finally reaches a value of 58.6 (45 °C). Given this significant increase, it can be concluded that adsorption is thermodynamically advantageous in these circumstances since higher temperatures significantly enhance contacts between Cd(ii) ions and the hydrogel's surface. Also, the *J* parameter that indicates the heterogeneity of energy sites increases from 0.024 to 0.092 then to 0.25.^41^ From the trend observed, at higher temperature values, the distribution of adsorption energies became wider. Such widening would generally mean either the presence or greater accessibility of an increased number of adsorption sites with different energy levels. This may result from structural changes or swelling processes in the hydrogel at higher temperatures. Generally speaking, data from the Jossens isotherm supports temperature's positive effect on both adsorption affinity and the number of energy levels in FAACP hydrogel, making this material more efficient for removing Cd(ii) over varying temperatures.

### 3.4. Adsorption kinetics

**Fig. 6:**
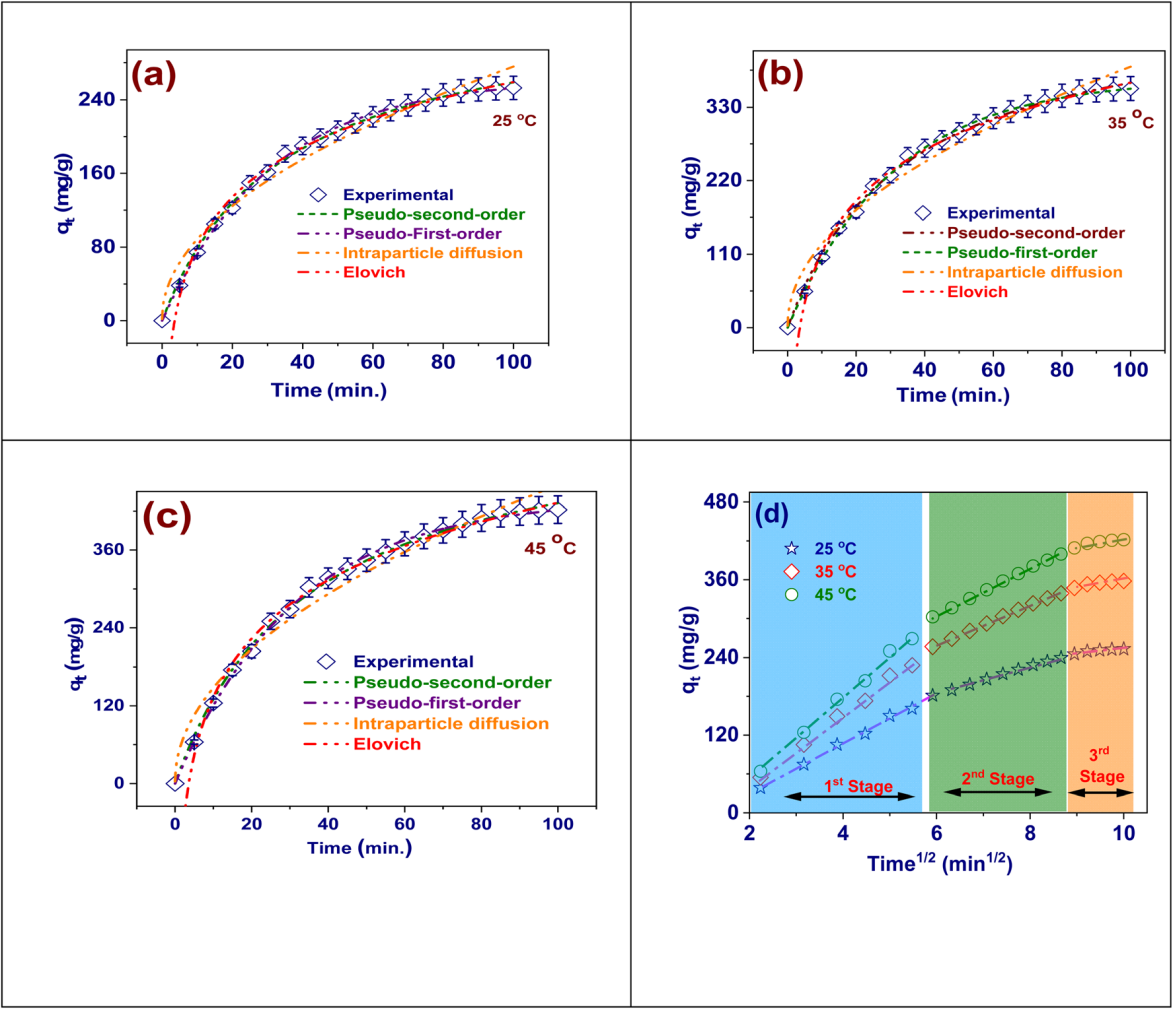
The adsorption kinetic models for adsorption of Cd(ii) onto FAACP beads at different temperature: (a) at 25 °C, (b) at 35 °C, (c) at 45 °C, (d) IPD model at 25 °C.

There are several benefits to using adsorption kinetic models to investigate the removal of Cd(ii) by FAACP beads since they offer a thorough examination of the rate, processes, and critical stages of the adsorption process. These models are essential for figuring out whether surface reactions, diffusion mechanisms, or a mix of both interactions are the main forces for adsorption.^42^ This understanding is important for maximizing contact time and developing effective water treatment methods. According to the pseudo-first-order model, the number of unoccupied adsorption sites closely correlates with the degree of adsorption. It is applicable in the initial stages of adsorption, when physical interactions predominate.^43^ But, as illustrated in Fig. 6(a–c), its importance reduces during the constant state. The model of pseudo-second-order suggests that the adsorption method is controlled by a chemisorption mechanism concerning interactions through allocation or transferring electrons. Because it can precisely forecast both the equilibrium adsorption capacity and kinetics for the full duration, this model generally describes the adsorption of Cd(ii) onto FAACP more accurately.^44^ The model of Intraparticle Diffusion will be used to check if the diffusion of Cd(ii) ions into the internal pores of the hydrogel is the step that limits the rate of adsorption. If it does not pass through the origin, this means that both surface adsorption and internal diffusion are involved in the overall procedure. The model of Elovich can apply for describing chemisorption processes on very heterogeneous surfaces like hydrogels. This model predicts that as time goes on with increasing saturation of surface concentration, adsorption rates decline.^45^ In conclusion, the application of such kinetic models offers profound knowledge regarding the dynamics involved in adsorption phenomena, which is important for optimizing the design, scaling-up, and presentation of Cd(ii)-removing hydrogel-based systems, as exposed in Table S6.

The pseudo-first-order adsorption model's kinetic limits are displayed in Table S8. This model is used to define how Cd(ii) adsorbs onto FAACP beads at three different temperatures.^42^ The model of pseudo-first-order is represented by the rate constant *k*_1_ (min^−1^ × 10^−2^), which describes the adsorption procedure as first-order kinetics. The number of vacant sites in this model closely correlates with the adsorption degree. For this study, the *k*_1_ value increased from 0.022 (25 °C) to 0.028 (35 °C) and additional to 0.032 (45 °C); thus, it indicates an increasing trend in the rate of Cd(ii) uptake with temperature elevation. This trend implies that more thermal energy improves the mobility of Cd(ii) ions toward their active binding sites on FAACP hydrogel in initial stages. Though this model adequately portrays early stages of an adsorption process, it underestimates fully developed capacity at equilibrium and does not provide a complete description of kinetic behaviors concerning chemisorption or multiple diffusion mechanisms. Therefore, while useful for understanding initial dynamics of adsorption and effects of temperature, the model of pseudo-first-order is typically applied alongside other models such as the model of pseudo-second-order for more inclusive insights into what kinetic phenomena are occurring.

Table S8 gives a summary of the parameters for the pseudo-second-order kinetic model concerning the adsorption of Cd(ii) on FAACP hydrogel beads at temperatures of 25, 35, and 45 °C. These results will be useful in evaluating the mechanism of chemisorption and the total efficiency of the adsorption process.^43^ The pseudo-second-order model suggests that the rate-limiting step of adsorption is associated with the bonding between the functional groups on the surface of beads and Cd(ii) ions through valence forces (*i.e.*, sharing or exchange of electrons). The values of rate constant (*k*_2_) decrease with increasing temperature as it varies from 8.34168 × 10^−5^ g mg^−1^ min^−1^ (25 °C) to 5.00099 × 10^−5^ (45 °C) indicating an exothermic reaction. This can be clarified by the detail that in this case, an adsorption reaction is being accelerated due to rising temperatures, but at the same time, the approach to equilibrium is decreasing slightly because saturation is beginning to occur on available adsorption sites. Results show a marked and consistent increase in equilibrium adsorption capacity (*q*_e_) which changes from 255.6 mg g^−1^ (25 °C) to 358.2 mg g^−1^ (35 °C) and finally reaches 422.4 mg g^−1^ (45 °C) suggesting that the adsorption method is endothermic and more promising by increasing temperature probably due to enhanced mobility of ions and better interaction within hydrogel matrix.

The parameters of the kinetic model of Intraparticle Diffusion for Cd(ii) adsorption on FAACP at 25, 35, and 45 °C have been presented in Table S7. The limits help analyze the internal mass transfer mechanisms in the system. The rate constant of intraparticle diffusion (*K*_*i*_) shows a clear increase with increasing temperature, from 27.38 mg g^−1^ min^−1/2^ (25 °C) to 38.74 (35 °C) and up to 45.66 (45 °C) (Fig. 6(a–c)). This trend confirms that higher temperatures favor the Cd(ii) ions diffusion into the internal cavities of the hydrogel due to enhanced ion mobility and reduced solution viscosity. The intercept (*X*), which represents boundary layer control, increases from 2.005 mg g^−1^ at lower temperatures to 3.34 mg g^−1^ at higher temperatures, indicating greater importance of surface adsorption at higher temperatures.^44^ The presence of non-zero intercepts displays that intraparticle diffusion is just part of a complicated adsorption device that also includes interactions on the surface and film diffusion. Therefore, it cannot explain the whole adsorption process. To sum up, the model points out the role of intraparticle diffusion at higher temperatures and agrees with the idea that Cd(ii) adsorption on FAACP is affected by internal diffusion along with other kinetic factors. Fig. 6(d) shows how intraparticle diffusion controls the adsorption of Cd(ii) ions onto FAACP at different temperatures. This is signified by a graph where adsorption capacity (*q*_e_) is plotted against the square root of time (*t*^1/2^). The results indicate that there are three distinct steps in the adsorption process, each one corresponding to a different stage of diffusion and happening sequentially. The first step, marked by an obvious linear rise in *q*_e_, indicates external surface diffusion or boundary layer transport. In this early stage, Cd(ii) ions quickly move from solution toward the outer surface of the adsorbent.^44^ The process under study shows a clear increase at higher temperatures which helps to increase the mobility of ions and increases the degree of mass transfer. The next phase, which is more gradual and straighter, relates to the mechanism of intraparticle diffusion. In this phase, ions enter the internal porous assembly of the hydrogel beads. This phase acts as a rate-limiting factor that is clearly accelerated at higher temperatures since an increase in thermal energy facilitates pore accessibility and activates more adsorption sites. The last phase shows a leveling trend indicating equilibrium, where active binding sites are filled up completely so that there is a fall in the adsorption rate. There is an obvious increase in adsorption capacity for all phases with temperature rise hence confirming the characterization of the overall process as endothermic. The three-phase behavior seen indicates that Cd(ii) are adsorbed onto FAACP beads through a multi-step diffusion mechanism with intraparticle diffusion being mostly responsible plus this behavior also indicates how temperature positively affects external and internal mass transfer processes.

The parameters of the kinetic model of Elovich applied to Cd(ii) adsorption on FAACP at 25, 35, and 45 °C are recorded in Table S8. The study's findings shed light on the material's surface heterogeneity and chemical adsorption properties. The Elovich model is applicable in cases where there is energetic heterogeneity on the adsorbent surface which leads to adsorption mechanisms governed by chemisorption. The coefficient *β* (g mg^−1^) is particularly significant as it indicates both surface coverage and activation energy pertinent to a chemisorption process.^45^ This parameter shows a large increase with an increase in temperature, from 77.89 (25 °C) to 110.22 (35 °C) and finally reaching 129.93 (45 °C). Such patterns would support the idea that higher temperatures enhance surface interactions and probably increase the number of active places obtainable for binding Cd(ii). On the other hand, the parameter *α* (mg g^−1^ min^−1^) which represents initial adsorption rate decreases by increasing temperature from 0.00358 (25 °C) to 0.00215 (45 °C) indicating that though total adsorption becomes more significant at higher temperatures very first stage of adsorption is taking place at lower rates due to intensified competition for high-energy binding sites during early stages of the adsorption process. These findings are in agreement with Elovich model that claims adsorption of Cd(ii) on FAACP happens through a chemisorption pathway marked by increased surface interactions with rising temperatures and highlighting even more how complex and varied the adsorbent surface is.

### 3.5. Adsorption thermodynamics

**Fig. 7:**
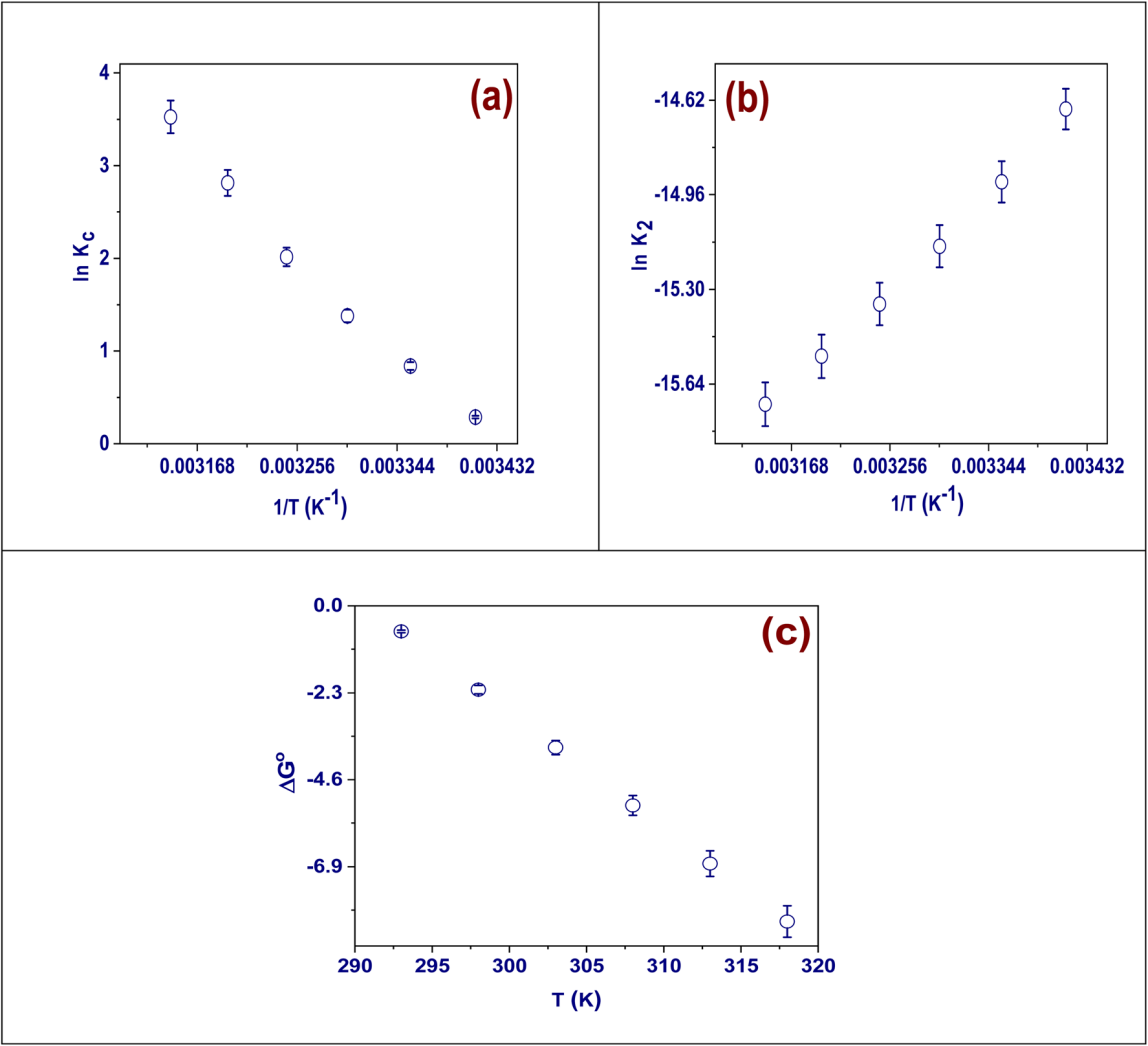
(a) Van't Hoff's plot, (b) the Arrhenius plot, and (c) temperature effect onto Δ*G*°.

The effects of temperature on the absorption of Cd(ii) ions between 293 and 318 K were thoroughly investigated using the provided formulas. Important variables like activation energy, variations in enthalpy, changes in Gibbs free energy, and changes in entropy were to be calculated for this investigation.^46^ The adsorption of Cd(ii) is spontaneous at all temperatures, as indicated by the negative values of Δ*G*°. Additionally, the absolute values of negative Δ*G*° grow significantly with temperature. This analysis establishes that such a trend observed here confirms an increase in entropy that favors the adsorption of Cd(ii), which makes it more spontaneous at higher temperatures (Fig. 7(a)). The entropy change (Δ*S*°) was calculated to be 306.95 J mol^−1^ K and represents increased disorder between solid and solution phases due to Cd(ii) adsorption (Fig. 7(b and c)). Furthermore, the change in enthalpy (Δ*H*°) will be used here to explain this interaction in more detail,^47^ the process under examination is endothermic, as indicated by the positive value of 89.25 kJ mol^−1^.^48^ A deeper look into the data in Table S9 reveals that using the Arrhenius equation gives an adsorption energy value of 42.6 kJ mol^−1^, as stated in Table S9. These results further confirm that the adsorption mechanism falls under chemisorption.

### 3.6. The mechanism of interaction

**Fig. 8:**
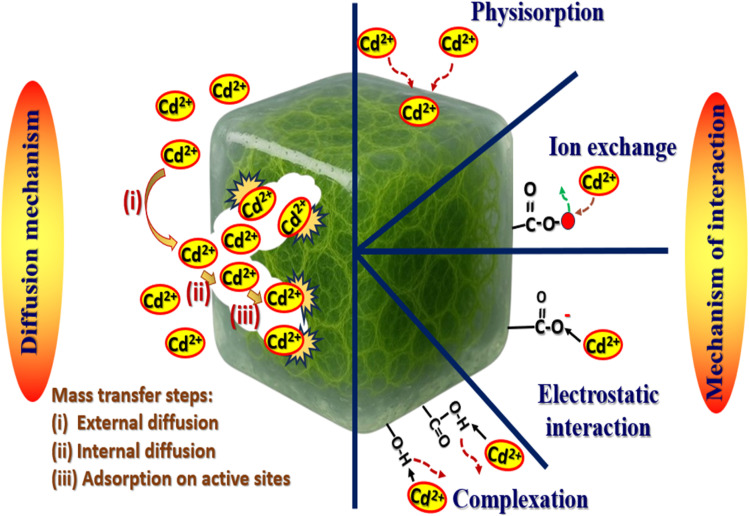
The interaction mechanism of Cd(ii) ions onto FAACP hydrogel beads.

The mechanism of Cd(ii) ions adsorption on FAACP hydrogel beads includes a series of important processes and interactions. This mechanism can be divided into some basic parts: at pH 6, the most important factor is electrostatic attraction because adsorption takes place with the surfaces gaining negative charge. The point of zero charge (pH_pzc_) has been originate to be 4.5. Therefore, positively charged Cd(ii) ions will be attracted to the negatively charged sites on the surface of the adsorbent material. The presence of useful groups in an adsorbent material increases its efficiency for adsorption, especially hydroxyl (–OH), amino (–NH_2_), and carboxyl (–COOH) groups. Additionally, FAAP hydrogel beads include metal centers that can form coordination bonds with Cd(ii) ions, strengthening the adsorption mechanism. Coordination complexes with Cd(ii) ions are facilitated by the incorporation of donor atoms, such as nitrogen and oxygen, into the structure of active algae. The present study involved an extensive analytical investigation of the adsorption process within a temperature range extending from 20 to 45 °C while strictly obeying to optimal conditions regarding the independent adjustable.^49^ The external mass transfer step is another name for this phase. Cd(ii) ions must pass through the hydrogel medium and the pores of the FAACP beads in order to reach the surface. The ease of this movement will depend on the size and form of the pores in FAACP beads. Mechanisms such as intraparticle diffusion and pore filling, which utilize preexisting voids as previously mentioned, would increase the entry of Cd(ii) ions into the pore structure of FAACP beads. This diffusion kinetics has a major role in determining the kinetics of the entire adsorption process. Cd(ii) ion adsorption on FAACP beads is a complicated process that involves a number of mechanisms, including diffusion kinetics, complexation events, and electrostatic attraction forces. External factors like pH and ionic strength have a significant impact on this process. As shown in Fig. 8, designing adsorbents for the removal of metal ions from aqueous solutions necessitates a deep comprehension of these essential elements.

### 3.7. Effect of salinity and interfering ions

**Fig. 9:**
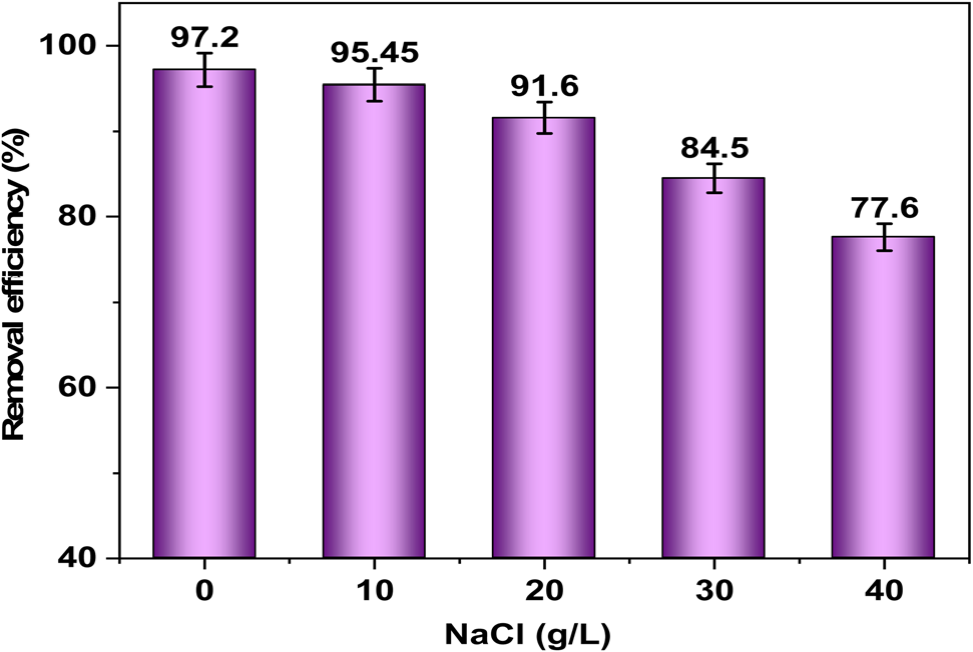
The impact of salinity on Cd(ii) ion adsorption and elimination onto FAACP beads.

NaCl was introduced to an ionic solution at concentrations of 10 to 40 g L^−1^ in the present work, to systematically study the effect of ionic strength on the Cd(ii) ions adsorption using FAACP beads. The data presented in Fig. 9 were analyzed and it was observed that with increasing ionic strength, there is a marked decrease in Cd(ii) removal efficiency, thus representative a strong association among ionic strength and adsorption efficacy. This change is noted as significant since it occurs at a Na^+^ concentration of 40 g L^−1^. These findings imply that electrostatic contact among Cd(ii) ions and FAACP are inhibited by the addition of NaCl. This also further supports the concept that chemical interactions exist between FAACP and Cd(ii) ions.^50^

The results in Fig. S1 specify that the adsorption efficiency of FAACP beads for Cd(ii) ions is greatly influenced by competing background ions present in contaminated water. In a 400 mg L^−1^ solution of Cd(ii) with 50 mg L^−1^ of interfering ions (25 mL volume, 100 min contact time, and at 25 °C), the capacity of adsorption was found to be 238.7 mg g^−1^ in the absence of any other ions. With the addition of monovalent cations such as K^+^ and Na^+^, it dropped slightly to 212.4 and 231.8 mg g^−1^, which means that there was not much competition for sites from these ions. However, divalent cations like Mg^2+^ and Ca^2+^ reduced this value more significantly down to 206.4 and 196.2 mg g^−1^ because they have stronger electrostatic attraction towards negatively charged groups within the hydrogel. Anions including Cl^−^, NO_3_^−^, PO_4_^3−^, and SO_4_^2−^ also lowered the uptake of Cd(ii) with capacities of 224, 239.4, 231.6, and 210.4 mg g^−1^, respectively probably due to ionic atmospheres or metal–anion complexes preventing access for Cd(ii). The large adsorption capacities observed here even in spite of these reductions underscore the high selectivity and efficiency that can be achieved by FAACP beads when removing Cd(ii) from multi-ionic wastewater systems; thus emphasizing their potential role in real complex wastewater systems having different dissolved metal ions.^51^

### 3.8. The effect on real water samples

The evaluation of the performance of extraction Cd(ii) ions from FAACP beads was carried out by experiments in authentic water media such as saline water, drinking water, and manufacturing effluent. Removal efficacy data were then collected in tap water (Table S10). The main purpose of this work was to determine wastewater and seawater removal efficacy. Results showed different removal rates for different types of assessed waters using a standard concentration of 100 mg L^−1^ of Cd(ii): wastewater with a removal efficacy of 91.2%, seawater with an efficacy of 82.6%, and tap water with a recorded removal rate at 96.2%. Data obtained up to now indicate that FAACP has high effectiveness towards elimination of Cd(ii) from diverse environmental contexts.^52^

### 3.9. Reusability

**Fig. 10:**
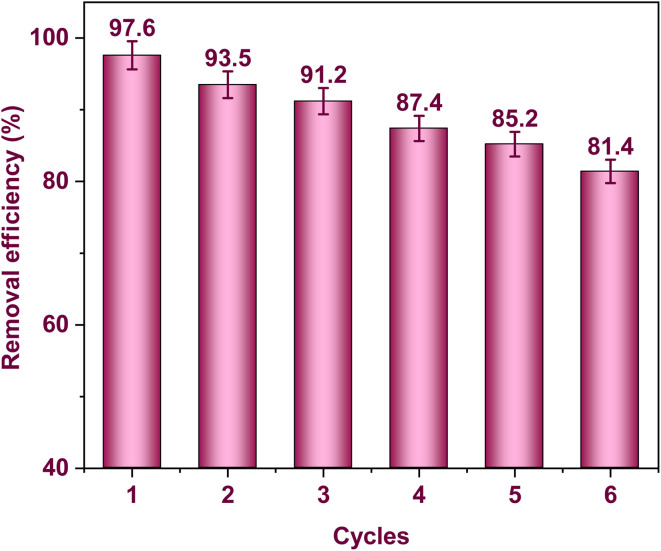
Efficacy of FAACP hydrogel beads regeneration.

The experiment was conducted with 50 mL of an aqueous solution containing 100 mg L^−1^ of Cd(ii) ions and 0.02 g of FAACP beads. The pH was adjusted to 6 and maintained constant over 100 min for the adsorption study of Cd(ii) ions. After adsorption, the filtrate containing the saturated sorbent was used to treat another 50 mL solution containing 0.05 mol L^−1^ ethylenediaminetetraacetic acid (EDTA). The mixture was kept at 298.15 K for four hours while being constantly stirred. This precise control aimed to maximize the interactions between the sorbent and EDTA, and then centrifugation was used to separate FAACP from the rest of the mixture. A washing step with bi-distilled water followed. The material was dried at 333 K for reuse in further cycles. Adsorption and regeneration processes took place six times, demonstrating good reactivation ability of sorbent an important criterion for commercial applicability. Considerable reduction in retention capacities occurred during six successive cycles of adsorption and desorption, with percentage values recorded at 97.6, 93.5, 91.2, 87.4, 85.2, and 81.4% of the initial capacity, as shown in this order (Fig. 10); such uptake decline is indicative of exhaustion of active sites plus changes in particular geometric arrangement of material intensified more with each additional cycle. These results indicate that the FAACP has great potential for practical recycling uses. The stability of the FAACP was assessed through XRD analysis after the regeneration process, as shown in Fig. S2.^53^

### 3.10. This adsorbent's comparison with others

Table S11 compares the adsorption performance of FAACP toward Cd(ii) ions with several powder-based adsorbents. The difficulties in comparing various adsorbents with distinct physical forms such as powders and hydrogels under particular experimental circumstances in each analysis are highlighted by this comparison.

### 3.11. Statistical analysis

#### 3.11.1. ANOVA

The ANOVA table gives a statistical view of the elements that have an impact on how Cd(ii) ions are adsorbed by FAACP beads. The results show that the overall model is highly significant (*F* = 50.82, *P* < 0.0001), which means that the factors considered in the model are important for the adsorption process. Specifically, individual variables pH (*A*), adsorbent quantity (*B*), and especially interaction time (*C*) have a large effect on the Cd(ii) removal with most influence from contact time (*F* = 309.66, *P* < 0.0001). The statistical analysis shows that communication among dose and time (*BC*) is noteworthy at *P* = 0.0132 while interactions *AB* and *AC* do not have noteworthy effects. The quadratic relationships for *A* (*A*^2^) and *C* (*C*^2^) are very significant indicating considerable curvature in their respective effects; however, the quadratic term for *B* (*B*^2^) is not significant. It indicates low residual error value of 1044.06 with 7 degrees of freedom. Also, since there is no pure error in the residual, it can be said that the fit may be very good or else there were not enough repetitions of experiments carried out. The model has a low standard deviation of 12.21 concerning adsorption performance prediction regarding Cd(ii) ions which means that there is less variation in data; plus with a mean value at 127.80 together with a coefficient variation at 9.56%, these statistical values mean that this model has high accuracy and reliability as further described in Table 2.^54^

**Table 2:** Examination of variance has been performed on the employed models

Source	Sum of squares	df	Mean squares	*F*-Value	*p*-Value	
Model	68218.96	9	7579.88	50.82	<0.0001	Significant
*A*-pH	1201.72	1	1201.72	8.06	0.0251	
*B*-dose	5012.44	1	5012.44	33.61	0.0007	
*C*-time	46185.93	1	46185.93	309.66	< 0.0001	
*AB*	176.05	1	176.05	1.18	0.3133	
*AC*	338.51	1	338.51	2.27	0.1757	
*BC*	1618.71	1	1618.71	10.85	0.0132	
*A* ^2^	4472.40	1	4472.40	29.99	0.0009	
*B* ^2^	20.26	1	20.26	0.1358	0.7234	
*C* ^2^	8354.57	1	8354.57	56.01	0.0001	
Residual	1044.06	7	149.15			
Lack of fit	1044.06	3	348.02			
Pure error	0.0000	4	0.0000			
Cor total	69263.02	16				
Std. Dev	12.21					
Mean	127.80					
C. V.%	9.56					
*R* ^2^	0.9849					
Adjusted *R*^2^	0.9655					
Predicted *R*^2^	0.7588					
Adeq precision	24.6153					

The statistical analysis in the table clearly shows the strength and reliability of the model proposed for the Cd(ii) adsorption onto FAACP beads. This model has an *R*^2^ value of 0.9849, which means that about 98.5% of the variation in Cd(ii) adsorption can be explained by it. The adjusted *R*^2^ value is 0.9655, further confirming the appropriateness of this model concerning its fit and taking into consideration how many independent variables were used; this value indicates only a slight drop from the original *R*^2^, typical for a well-fitting model.^55^ The predicted *R*^2^ value of 0.7588 is lower, yet it still reflects a relatively good predictive capacity. The difference between this figure and the adjusted *R*^2^ presents room for some enhancements in the model or may even indicate overfitting. The precision metric is recorded at 24.6153, which substantially surpasses the benchmark threshold of 4 and thus reveals an excellent signal-to-noise ratio; it further corroborates that this model describes adequately conditions to permit efficient exploration within the design space. This also means that this model has a high precision and consistency in describing how Cd(ii) adsorbs onto FAACP hydrogel beads since it has a low standard deviation of 12.21, a high mean response of 127.80, and a low coefficient of variation (C. V.% = 9.56%) as accessible in Table 2.

Table of model summary statistics presents a comparative analysis of four modeling approaches: linear, 2FI, quadratic, and cubic models. The adequateness of these models in telling the Cd(ii) ions adsorption behavior on FAACP beads is discussed. The quadratic model is declared as the best fitting model since it has a low sequential *p*-value (0.0002), indicating high statistical significance. It also has the highest adjusted *R*^2^ value (0.9655) along with a good predicted *R*^2^ value (0.7588), which will suggest not only a good fit to experimental data but substantial predictive reliability. On the other hand, both linear and 2FI models have higher mean square values but lower adjusted *R*^2^ values (0.7004 and 0.6597) with less predictive capability as indicated by their predicted *R*^2^ values (0.5874 and 0.3107). This means these models are not so good in capturing accurately the dynamics of this particular system under study. Although cubic gives an adjusted *R*^2^ of 1.0000, it is aliased; hence results will be affected by confounding factors in the experiment set up and cannot be considered reliable for prediction purposes. Therefore, statistical analysis strongly supports quadratic as the best fitting model for explaining and predicting adsorption efficiency for Cd(ii) based on parameters from Table 3.^56^

**Table 3:** Sum of squares for successive models

Source	Sum of squares	df	Mean square	Sequential *p*-value	Adjusted *R*^2^	Predicted *R*^2^	
Linear	16862.93	9	1873.66	0.0003	0.7004	0.5874	
2FI	14729.66	6	2454.94	0.7016	0.6597	0.3107	
Quadratic	1044.06	3	348.02	0.0002	0.9655	0.7588	Suggested
Cubic	0.0000	0			1.0000		Aliased

The sum of squares table for the sequential model compares the variability in the Cd(ii) adsorption onto FAACP beads explained by the different complexities of models. It starts by looking at the mean against the total, which gives a significant sum of squares, 2.776 × 10^5^ indicative of total data variability. The linear model accounts for a considerable part of this with a sum of squares equal to 52400.09 and an *F*-value equal to 13.47 and *p*-value significant at 0.0003 indicating that it substantially improves the model's ability to explain results. Moving on to the two-factor contact (2FI) model does not show much better fit since its *F*-value was only 0.4828 with a *p*-value of 0.7016; this means these interactions have little effect on the outcome. On the other hand, there is very strong evidence that the model of quadratic fits well than the 2FI model since it has an *F*-value equivalent to 30.59 and a *p*-value equal to 0.0002—thus supporting quadratic terms included as best fitting. The cubic model is not reliable because it is aliased with the quadratic model due to confounding factors; hence, results cannot be trusted. A zero-residual sum of squares can indicate either perfect fitting or overfitting in addition to data absence. In general terms, though, the quadratic model would be preferred for predicting Cd(ii) adsorption on FAACP beads (Table 4).^57^

**Table 4:** Sequential model of sum of squares

Source	Sum of squares	df	Mean square	*F*-Value	*p*-Value	
Mean *vs.* total	2.776 × 10^5^	1	2.776 × 10^5^			
Linear *vs.* mean	52400.09	3	17466.70	13.47	0.0003	
2FI *vs.* linear	2133.27	3	711.09	0.4828	0.7016	
Quadratic *vs.* 2FI	13685.60	3	4561.87	30.59	0.0002	Suggested
Cubic *vs.* quadratic	1044.06	3	348.02			Aliased
Residual	0.0000	4	0.0000			
Total	3.469 × 10^5^	17	20406.52			

The equation obtained in terms of coded factors is applied for forecasting the adsorption capacity (*q*_e_) of Cd(ii) onto FAACP beads at varying levels of pH (*A*), adsorbent quantity (*B*), and interaction time (*C*). In this equation, all factors are expressed in coded units where +1 and −1 represent the high and low levels of each factor, individually. The use of coded units helps to conveniently compare not only the magnitudes but also the signs of the coefficients involved, thus allowing a relative effect assessment. The positive coefficient for contact time implies that longer times are favorable for adsorption, while the negative coefficient for dosage indicates that beyond an optimal value, increasing the dosage might reduce efficiency.^58^ The presence of interaction terms (*AB*, *AC*, and *BC*) indicates that the interaction between two variables together affects the response. The attendance of quadratic terms (*A*^2^, *B*^2^, and *C*^2^) indicates a nonlinear response and suggests the existence of an optimum condition rather than a linear relationship. This coded equation is an essential tool for studying system dynamics, simplifying optimization processes, and facilitating the identification of the most significant factors and their interactions concerning Cd(ii) removal efficiency as described in eqn (5):

5 *q*_e_ = 165.129 + 12.2562 × *A* − 25.0311 × *B* + 75.9819 × *C* − 6.63425 × *AB* + 9.1993 × *AC* − 20.1166 × *BC* − 32.5913 × *A*^2^ − 2.19343 × *B*^2^ − 44.5445 × *C*^2^

The actual equation that describes the Cd(ii) ions adsorption onto FAACP beads in real and exact units for the significant factors: pH, adsorbent dosage (in grams), and contact time (in min). This equation will be employed to make a more detailed prediction of the adsorption capacity (*q*_e_) under certain experimental situations. The coded equation intends to normalize variables for comparison but keeps the actual equation with all variables in their original scales. Therefore, any predictions made using this equation will require real measurements of pH, dosage, and time never standardized values. It is thus suited to practical use as well as easy modification in an experimental context but cannot be used for testing relative importance among factors. This limitation results from different scales of coefficients with measurement units for each variable; moreover, the intercept indicates a response at some location in the design space rather than its center. Even though such an effect can have a beneficial prediction, it cannot inform about how large effects are with respect to particular factors or their interactions, as mentioned in eqn (6):

6 *q*_e_ = −78.8019 + 39.3045 × pH + 54.219 × Dose + 3.80862 × Time − 9.21424 × pH Dose + 0.0645565 × pH Time − 1.76461 × Dose Time + −3.62126 × pH^2^ − 38.0803 × Dose^2^ − 0.0197427 × Time^2^

**Fig. 11:**
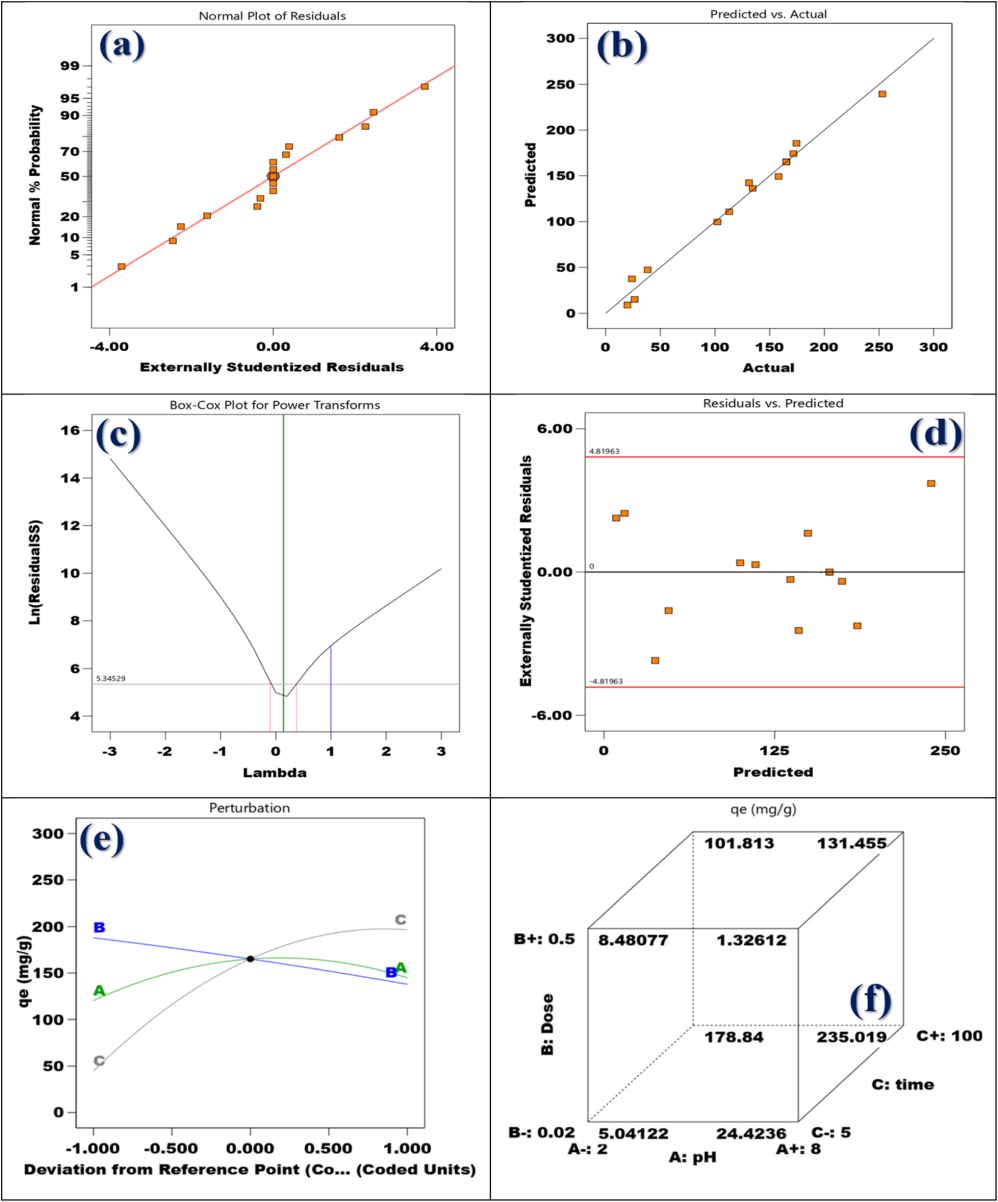
(a) Plotting the correlation among normal% probability, (b) predicted *vs.* actual, (c) the Box–Cox plot for Cd(ii) power transformations, (d) highly normalized residuals compared to predictions, (e) perturbation plot, and (f) cubic communication.

As a visual aid for determining whether the residuals in the regression model concerning Cd(ii) adsorption onto FAACP beads are normal, Fig. 11(a) displays a normal probability plot of externally studentized residuals. In this plot, orange squares are used to depict individual observations, while the red line indicates what would be expected if residuals follow a normal distribution. Most observations fall close to the red line, which means that residuals probably have a normal distribution and thus fulfill an important assumption required for both ANOVA and regression analysis. It also means that errors in the model are random and not biased this makes any statistical results based on the model more valid and reliable. Additionally, the lack of significant outliers or significant differences supports the claim that the model faithfully captures the results of the experiment.^58^

Fig. 11(b) presents a comparative study of the predicted and experimental values for the adsorption capacity of Cd(ii) onto FAACP beads. This comparison is essential for evaluating the accuracy and reliability of the regression model developed. The orange squares in this figure represent individual data points where the predicted outputs from the model are compared with those obtained from experiments. The solid diagonal line in this plot represents an ideal situation in which predicted values would exactly equal those observed experimentally. Close clustering of data points around this line confirms that model predictions are accurate, indicating high correlation and agreement between expected outcomes and actual results plus no significant discrepancies or repeating patterns further validate such a model as capable of capturing inherent relationships between experimental variables and adsorption response.^58^

The Box–Cox plot for influence transformation is presented in Fig. 11(c). This is a diagnostic plot that helps to regulate whether there is a need to transform the response variable so that it meets assumptions of normality and constant variance in the regression model for Cd(ii) adsorption onto FAACP beads. The residual sum of squares' natural logarithm (Ln (Residual SS)) is plotted on the *y*-axis, while values on the *x*-axis represent possible transformations by varying the parameter Lambda (*λ*). The green line at *λ* = 1 specifies no transformation has been applied – data used are in their original form. The point where this curve attains its minimum value gives us an optimal lambda that would reduce residual variance most effectively; here, it happens to be very close to 1 and falls between two other lines (red and blue) which define a 95% confidence interval around best-fit lambda. No transformation will therefore be required; hence, normality and homoscedasticity assumptions are satisfied for this model since proper scaling of the response variable has been achieved.^58^

Fig. 11(d) displays the residuals *vs.* fitted plot, which is a primary diagnostic device for checking the assumption of constant variance (homoscedasticity) and identifying any unusual patterns or outliers in the predictive model concerning the Cd(ii) adsorption on FAACP beads. This graphic compare predicted outcomes on the *x*-axis with externally simulated residuals on the *y*-axis. The best-case scenario is random scatter of residuals around zero with no discernible pattern; this condition is mostly confirmed by the figure. Horizontal red lines at ±4.82 establish control limits for identifying data points that may be considered outliers or highly influential observations. This study shows that every data point fall within these bounds, demonstrating that the model's assumption of constant variance is supported by the absence of significant outliers. Also, an even spread of residuals over the predicted range means that model ability to predict results is well maintained across its entire design space.

The perturbation plot is shown in Fig. 11(e) for the adsorption capacity of Cd(ii) on FAACP beads. The effects of pH (*A*), dose (*B*), and time (*C*) are shown with the other variables held constant. Predicted *q*_e_ values are shown in mL g^−1^ on the *y*-axis and deviations from a central point are noted in coded units (−1 to +1) on the *x*-axis. The gray curve for contact time (*C*) has very strong upward curvature which means that greater contact times increase Cd(ii) adsorption significantly and confirms this variable as being most influential. The green curve for pH (*A*) has an upward but less steep curvature which means that increasing pH will increase *q*_e_ slightly. The blue curve for adsorbent dose (*B*) displays slightly negative slopes indicating that increasing amounts of adsorbent may reduce adsorption slightly due to aggregation or saturation at high doses. This black dot in the center stands for a reference point and makes it easy to see how each variable influences the system; therefore, one can easily say that interaction time is an significant factor followed by pH and then adsorbent dose having almost no negative effect on Cd(ii) uptake.^58^

The three-dimensional interaction plot, as shown in Fig. 11(f), demonstrates the joint influence of pH (*A*), adsorbent amount (*B*), and contact time (*C*) on the capacity of adsorption (*q*_e_) of Cd(ii) ions onto FAACP beads. The cube corners depict the specific combinations of high and low levels for these three parameters, with respective *q*_e_ values in mg g^−1^ indicated at each vertex. The results reveal that under conditions at pH (8), very low adsorbent dosage (0.02 g), and long interaction time (100 min), maximum capacity of adsorption reaches a value of 235.019 mg g; this set condition proves to be most promising for the Cd(ii) ions adsorption. Conversely, minimum adsorption capacity found was 1.32612 mg g^−1^ when using more concentrated adsorbent (0.5 g) while maintaining high pH (8) but reducing contact time down to just five minutes.^58^ It can be concluded from this observation that excess adsorbent material with a short interaction time has a negative effect on the efficiency of the adsorption method. The analysis further revealed that contact duration has the greatest positive effect on the quantity of adsorbed metal ion Cd(ii) (*q*_e_) and next to it is pH. Increasing adsorbent concentration generally leads to a decrease in adsorption efficiency. This graph supports results from regression analysis and ANOVA by providing an in-depth insight into optimal conditions required for improved adsorption of Cd(ii) ions.

#### 3.11.2. Analyzing reaction surfaces and simulating experimental designs

**Fig. 12:**
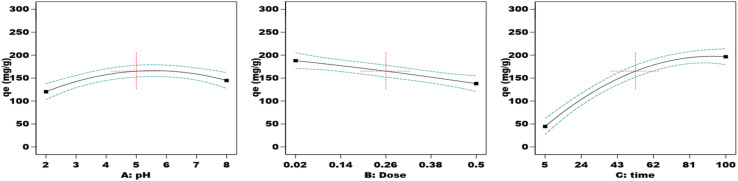
Effects of different adsorption conditions on the elimination of Cd(ii) ions: (A) solution pH, (B) adsorbent dose, and (C) contact time.

Three significant effects plots are depicted in Fig. 12, which represent the direct effects of pH (*A*), adsorbent amount (*B*), and contact time (*C*) on the capacity of adsorption (*q*_e_) of Cd(ii) ions onto FAACP beads. The first plot is a quadratic effect with pH; *q*_e_ increases with pH values up to about 5 and slightly decreases at higher pH values thereafter. This means that an intermediate level of pH will be favorable for adsorption due to enhanced electrostatic attraction among Cd(ii) ions and the hydrogel surface. The second plot is a strong negative linear relationship with adsorbent dosage since it increases from 0.02 to 0.50 g, wherein mass-specific quantity adsorbed (*q*_e_) continuously falls. This may be due to the agglomeration of adsorbent particles or covering adsorption places, which reduce the active surface area available for adsorption. The third plot shows significant positive curvature concerning contact time, indicating large increases in *q*_e_ with contact time, particularly between 5 and 100 min. This trend confirms that increased contact time enhances adsorption by facilitating more interactions among Cd(ii) ions and the beads. Therefore, the most important factor affecting *q*_e_ is contact duration, followed by pH; adsorbent dose has a negligible negative impact.^59^

#### 3.11.3. Checking the model's sufficiency

**Fig. 13:**
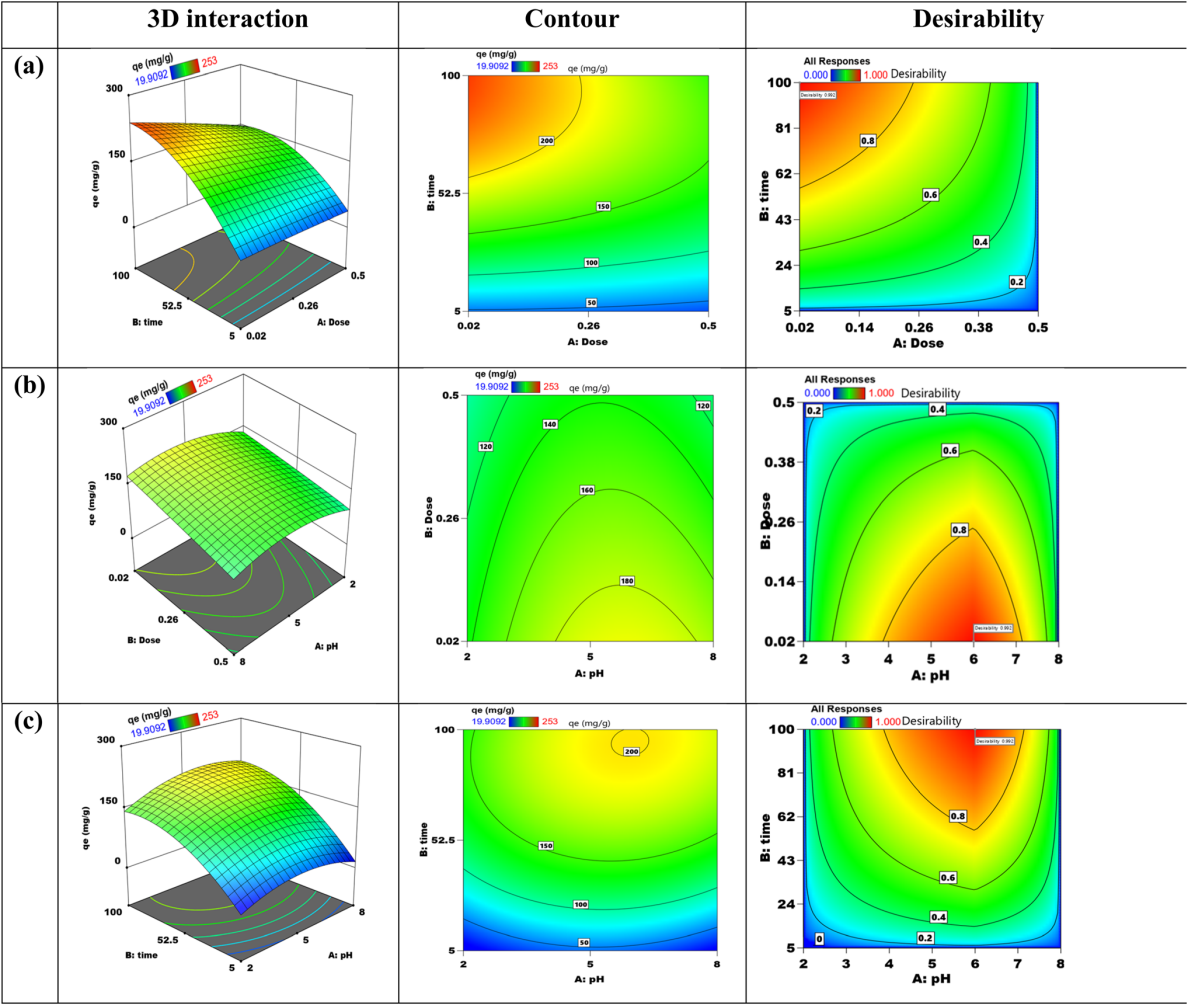
Contour and three-dimensional interaction among FAACP beads and Cd(ii) ions (a) dose and time, (b) pH and dose, and (c) pH and time.

Three views—a three-dimensional interaction plot, a contour plot, and a desirability plot—graphically depict the combined effects of adsorbent dosage (*A*) and contact time (*B*) on the adsorption capacity (*q*_e_) of Cd(ii) onto FAACP beads (Fig. 13(a)). The three-dimensional plot reveals that *q*_e_ increases considerably with increasing contact times but decreases slightly when higher adsorbent dosages are applied. This is evidenced by the color gradient changing from blue (indicating low *q*_e_ of around 19.9 mg g^−1^) to red (indicating high *q*_e_ of about 253 mg g^−1^); thus, longer contact durations at lower doses should be used for optimal adsorption. Such an observation is further validated by the contour plot which provides levels of *q*_e_ through curved lines and describes optimal adsorption as located in the upper-left quadrant with lower dosages and longer timeframes as indicated by gradients moving toward red. The desirability plot relates experimental conditions to high adsorption efficiency on a scale from 0 (least desirable in blue) to 1 (most desirable in red). Maximum desirability area ≈0.992 falls within low adsorbent dosages and long contact times, thereby supporting previous findings. All these visualizations confirmed that increasing contact time while decreasing adsorbent dosage enhances the removal efficiency of Cd(ii).

The relationship among pH (*A*) and adsorbent quantity (*B*) with respect to the adsorption capacity (*q*_e_) of Cd(ii) onto FAACP beads (Fig. 13(b)). Three different graphical representations are employed: a 3D interaction plot, a contour plot, and a desirability plot. The left panel shows a 3D interaction plot where the adsorption capacity (*q*_e_) rises with pH up to about 5 and at high adsorbent doses slightly decreases adsorption, creating a curved surface with maximum at the optimal pH and lower dosages. The color gradient from blue to red indicates increasing adsorption capacity reaching an approximate peak value of 253 mg g^−1^. A contour plot located at the center provides two-dimensional view concentric curves where peak *q*_e_ values appear at intermediate pH values and lower dosages confirming that these parameters interact synergistically. A desirability plot on the right suggests optimal conditions for maximum response levels; here, red-highlighted high desirability score area of about 0.992 is concentrated between pH 5 and 6 by an adsorbent quantity nearing 0.02 g. As either the dosage becomes more or as the pH approaches extremes there is a significant drop in desirability. All these graphs together provide strong proof that best adsorption of Cd(ii) happens when moderate pH and low adsorbent dosage conditions are applied supporting earlier statistical tests and theoretical models.^59^

Fig. 13(c) presents three complementary views: 3D interaction, contour, and desirability plots illustrating the interactive effects of pH (*A*) and contact duration (*B*) on adsorption capacity (*q*_e_) of Cd(ii) onto FAACP beads. A clear peak in *q*_e_ is seen at intermediate pH values (roughly between 5 and 6) and long contact times on the left-hand side in a 3D interaction plot indicating a very strong synergistic effect between these two factors. The values of *q*_e_ increase substantially from about 20 mg g^−1^ (in blue) to more than 250 mg g^−1^ (in red), which means it works well under certain combined conditions. This is corroborated by the central contour plot, which shows the highest *q*_e_ values in the higher central region where pH and contact duration are maintained at acceptable ranges. Additionally, optimization findings with a peak desirability score of 0.992 in the same moderate pH and extended contact duration area highlighted in red are displayed in the desirability plot on the right. The data shown in these plots suggest that the adsorption method of Cd(ii) is notably enhanced when the pH is kept close to the middle point of the range studied and enough time is given for interaction to take place; thus emphasizing an imperative need for its concurrent optimization to attain maximum possible removal efficiency.

#### 3.11.4. Model validation and the desirability approach

**Fig. 14:**
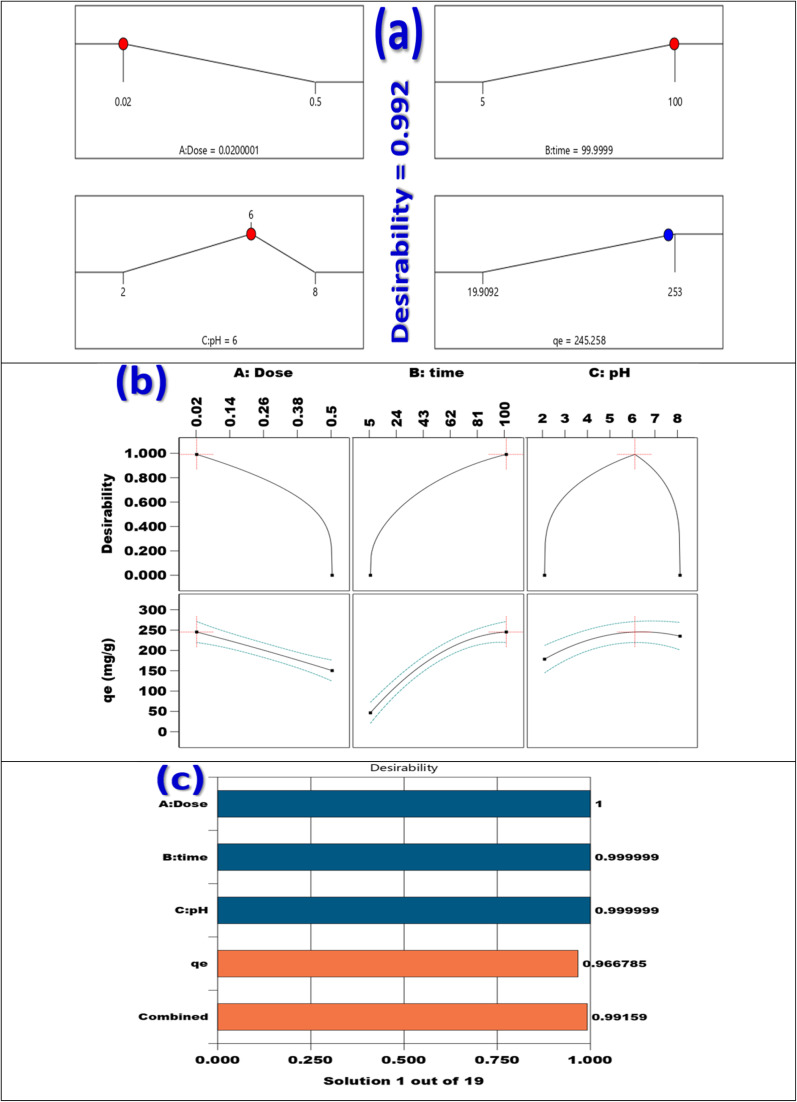
(a) Increasing curiosity about the numerically optimal solutions, (b) desirability of every response, and (c) bar graph representation of individual desirability.

A desirability optimization plot for the experimental settings to maximize the adsorption capacity (*q*_e_) of Cd(ii) ions onto FAACP beads is shown in Fig. 14(a). There are highly effective conditions because the analysis yielded a desirability value of 0.992, which is extremely near to its theoretical maximum value of 1.0. The findings show that the ideal quantity of adsorbent is at its lowest value of 0.02 g, suggesting that lower amounts improve process efficiency because there would be less particle aggregation and more surface accessibility. The optimal values for the operating parameters were determined in order to achieve the highest possible adsorption capacity for Cd(ii). The results presented that an optimal dosage of 0.2 g of adsorbent was required, indicating that a smaller amount of adsorbent is more effective in achieving maximum adsorption. A contact time of 100 min was also found to be optimal, suggesting that more time is essential to reach adsorption equilibrium among the adsorbent and Cd(ii) ions. The pH value of about 6 was identified as moderate for achieving good electrostatic interaction between FAACP and Cd(ii) ions. Under these conditions, an estimated adsorption capacity of 245.258 mg g^−1^ was obtained, which is close to the theoretical maximum value for this system (253 mg g^−1^). These results clearly demonstrate that low concentration dosage, moderate pH, and long contact time together play a significant role in maximizing the adsorption efficiency and, therefore, are very important parameters in developing a successful strategy for removing Cd(ii).^59^

The optimization plots accessible in Fig. 14(b) reflect the individual impacts of parameters like adsorbent quantity (*A*), interaction time (*B*), and pH (*C*) on the desirability as well as the adsorption capacity (*q*_e_) of Cd(ii) onto FAACP hydrogel beads. The upper part contains desirability curves for each factor showing how much closer those settings get to an optimal effect (value = 1). From this study, maximum desirability was found at a low dose of 0.02 g, long contact time of 100 min, and neutral pH of 6; hence these values are taken as optimal conditions. The lower part corresponds to plots of *q*_e_ against each factor with confidence intervals added for more clarity about their interrelationship with adsorption capacity. From this analysis, when increasing the effect of dose on *q*_e_, it can be seen that increasing dosage gives rise to a regular decrease in *q*_e_; this is probably due to site aggregation or saturation effects. Contact time shows very significant positive relationships since *q*_e_ increases markedly with its duration which means that as contact time increases both diffusion rates and surface interactions increase substantially. The pH level shows a quadratic relation with maximum efficiency at pH 6 decreasing at both lower and higher levels indicating that moderately acidic conditions favor optimum ion exchange efficiency. These findings further support the conclusion that optimum removal of Cd(ii) occurs under conditions of minimum dosage maximum contact time and moderate acidity which together maximize adsorption capacity and come close to achieving perfect model desirability.

Fig. 14(c) is the desirability bar chart that summarizes the optimization results for the adsorption of Cd(ii) onto FAACP hydrogel beads. It clearly illustrates how well each individual factor and the entire system conform to the required optimal conditions. The specific desirability scores are given here for input variables: *A* (dose) has an optimal score of 1.000, while *B* (time) and *C* (pH) very closely approach this ideal, with scores of 0.999999 each; these levels for experimental factors significantly enhanced the adsorption response and thus proved strong agreement with theoretical optimization parameters. The output variable *q*_e_ possesses a high desirability score, *i.e.*, 0.966785, which indicates that expected adsorption is very much in line with its theoretical maximum value. A cumulative desirability score of 0.99159 has been recorded for this particular configuration described as one among 19 evaluated scenarios; it can thus be claimed to be the best and most optimally balanced arrangement with respect to such performance. This analysis can support the claim that an extremely good set of operating conditions improving Cd(ii) removal efficiency was found by the model fulfilling all imposed experimental criteria.^59^

## 4. Conclusion

The current study presents the synthesis of an activated bio-adsorbent material from algae-hyaluronic acid activation through functionalization with glutamic acid. After functionalization, the algae were incorporated into a composite system of β-cyclodextrin and polyethylenimine, which cross-linked with epichlorohydrin to form FAACP hydrogel beads. These beads can be used in environmental applications since they demonstrated a high level of efficiency in eliminating Cd(ii) ions from effluent. The characterization of the beads was carried out using FTIR, XRD, XPS, SEM mapping, EDX, and surface area measurements by nitrogen adsorption/desorption isotherms. The bio-adsorbent has a surface area of 128.734 m^2^ g^−1^. Additionally, it examined how several factors including temperature, pH, dose, and beginning concentration affected the adsorption process. The findings demonstrated that the adsorption process fit the Langmuir isotherm well and obeyed the pseudo-second-order kinetic model; chemisorption was identified as the primary mechanism of adsorption having an energy required of 30.18 kJ mol^−1^. The findings also showed that metal adsorption rose with temperature, demonstrating the endothermic and spontaneous nature of this process. Analytical tests showed that using the Box–Behnken design with response surface approach using Design-Expert software significantly improved the adsorption procedure's performance. This investigation focused on certain parameter settings: applying 0.02 g of FAACP hydrogel beads per 25 mL solution at an optimum pH value equal to 6 and achieving an adsorption capacity equal to 254.75 mg g^−1^ for Cd(ii) removal from solutions. According to findings from data, the adsorbent demonstrated great stability and was very effective at repeatedly eliminating contaminants throughout six consecutive cycles of adsorption desorption.

## Author contributions

Ahlem Guesmi: conceptualization, data curation, investigation, validation, visualization, writing-review and editing. Naoufel Ben Hamadi: conceptualization, data curation, investigation, resources, validation, visualization, writing-review and editing. Wesam Abd El-Fattah: conceptualization, data curation, investigation, visualization, writing-review and editing. Mohamed G. El-Desouky: conceptualization, data curation, investigation, methodology, validation, visualization, writing-original draft. Ashraf A. El-Bindary: conceptualization, data curation, investigation, resources, validation, visualization, writing-original draft, writing-review and editing.

## Conflicts of interest

There are no conflicts of interest to declare.

## Supplementary Material

RA-016-D6RA00411C-s001

## Data Availability

The data that support the findings of this study are available from the corresponding author upon reasonable request. Supplementary information (SI) is available. See DOI: https://doi.org/10.1039/d6ra00411c.
