# Supplementary material for: Sustainable β-cyclodextrin/polyethylenimine-encapsulated activated algae hydrogel beads for high-capacity Cd(ii) removal: adsorption performance, mechanism, thermodynamics, and Box–Behnken optimization
Source: RSC Adv. 2026 Apr 7;16(20):17918–45. doi: 10.1039/d6ra00411c (PMC13054488; doi:10.1039/d6ra00411c)
Supplement: RA-016-D6RA00411C-s001 [file RA-016-D6RA00411C-s001.pdf]

Supplementary Information (SI) for RSC Advances.
This journal is © The Royal Society of Chemistry 2026

**Sustainable β-Cyclodextrin/Polyethylenimine-Encapsulated Activated Algae Hydrogel Beads for High-Capacity Cd(II) Removal: Adsorption Performance, Mechanism, Thermodynamics, and Box–Behnken Optimization**

Table S1. Chemical name, formula, and company.

| **Chemical name** | **Formula** | **Company** |
|---|---|---|
| Polyethylenimine | $C_{24}H_{63}N_{13}$ | Sigma-Aldrich, Germany |
| β-Cyclodextrin | $C_{42}H_{70}O_{35}$ | Sinopharm Chemical Reagent Co., Ltd, China |
| Epichlorohydrin, 99% | $C_3H_5ClO$ | Thermoscientafic |
| Algae | | Beach of mediterinane sea |
| Cadmium acetate | $Cd(CH_3CO_2)_2(H_2O)_2$ | Sigma-Aldrich, Germany |
| Sodium hydroxide | NaOH | LOBA CHEMIE PVT.LTD, India |
| Hydrochloric acid 37% | HCl | LOBA CHEMIE PVT.LTD, India |
| Ethanol | $C_2H_5OH$ | LOBA CHEMIE PVT.LTD, India |
| Methanol | $CH_3OH$ | LOBA CHEMIE PVT.LTD, India |

Table S2. Instruments and equibments.

| Test name | Abbrevation | Instrument name | Company | Illustration |
|---|---|---|---|---|
| Fourier transformer infrared | FT-IR | A Nicolet IS10 Fourier transform infrared (FTIR) spectrometer | Thermo Fisher Scientific, Waltham, MA, USA | equipped with an attenuated total reflectance accessory and which ran in the 4000-400 $cm^{-1}$ range was used to gather FTIR spectra |
| Powered X-ray diffraction | PXRD | Siemens diffractometer (model D500, Germany) | Germany | patterns were captured from powder samples through the use of a Siemens diffractometer (model D500, Germany) that was fitted with a Cu-K radiation source (wavelength 1.54 Angstroms (Å)) operating at 30 kV and 20 mA. |
| Scanning Electron Microscope | SEM | (JSM-6510LV, JEOL Ltd., Tokyo, Japan) | JEOL Ltd., Tokyo, Japan | The morphology of the investigated sorbents was analyzed with the use of a scanning electron microscope |
| X-ray photoelectron spectroscopy | XPS | K-ALPHA (Themo Fisher Scientific, USA) | Themo Fisher Scientific, USA | Used for determination the elemental analysis for the compound |
| Braunnar Emmet Teller | BET | Quantachrome Instruments, Anton Paar Quanta Tec, Inc., Boynton Beach, FL, | Quanta Tec, Inc., Boynton Beach, FL, USA | was utilised for surface and pore analysis (Brunauer Emmett-Teller (BET) surface area, porous volume, and pore size), and NovaWin Software (v11.0) was used for data interpretation. |

| | | | | |
|---|---|---|---|---|
| | | USA | | The BET surface area of material adsorbents was obtained by the application of nitrogen adsorption-desorption isotherms at 77K through the use of a specific analyser (Quadrasorb–EVO, Quantachrome, USA). |
| Atomic adsorption spectrometer | | PerkinElmer atomic absorption spectrometer (PinAAcle 500), Singapore | PerkinElmer, Singapore | Measuring the metal ion concentration. |
| Energy Dispersive X-ray | EDX | Leo1430VP microscope | Carl Zeiss AG, Jena, Germany | Elemntal analysis of the material |
| Transmission electron microscopy | TEM | TEM, FEI Teanci G2 F20, USA | FEI Teanci G2 F20, USA | Determination the morphology of the material and size |
| pH meter | pH | HANNA (model 211) | USA | Measuring the acidity or basicity of the solution |
| Sonication | Ultrasonic | Elmasonic P300H ultrasonic bath, continuous mode, power 380 W | Elma Schmidbauer GmbH, Singen, Germany | Sonication of the material as well as used ton disperse material on the solution as it decrease the particle size of the material |
| Water bath | Shaking | GFL Orbital Shaker 3017 | | |

Table S3. True variables for the adsorption process factors.

| Factors | pH | Time (min.) | Initial concentration (mg/L) | Temprature ($^oC$) | Dose (g) |
|---|---|---|---|---|---|
| pH | 2-8 | 30 | 200 | 25 | 0.02 |
| Dose (g) | 6 | 30 | 300 | 25 | 0.02-0.5 |
| Initial concenteration | 6 | 100 | 20-380 | 25 | 0.02 |
| Time (min.) | 6 | 5-100 | 300 | 25-45 | 0.02 |
| Temperature | 6 | 30 | 300 | 20-45 | 0.02 |

Table S4. True variables, codes, and their BBD levels.

| Code | Variables | -1 | 0 | +1 |
|---|---|---|---|---|
| A | pH | 2 | 7 | 12 |
| B | Dose (g) | 0.02 | 0.26 | 0.5 |
| C | Time (min.) | 5 | 55.5 | 100 |

Table S5. Crystalographic data.

| *hkl* | 2θ Observed | 2θ Calculated | Difference |
|---|---|---|---|
| -1 2 1 | 12.5281 | 12.4945 | 0.0336 |
| 1 1 1 | 17.1265 | 17.0705 | 0.056 |
| -2 0 0 | 18.6877 | 18.6408 | 0.0469 |
| -2 2 0 | 20.3658 | 20.3251 | 0.0407 |
| 0 5 1 | 22.5096 | 22.4675 | 0.0421 |
| -1 4 2 | 23.5758 | 23.525 | 0.0508 |
| -1 5 2 | 26.5393 | 26.5139 | 0.0254 |
| 2 3 1 | 28.0104 | 27.9779 | 0.0325 |
| -3 1 0 | 28.454 | 28.4159 | 0.0381 |
| -3 2 0 | 29.2759 | 29.289 | -0.0131 |
| -3 2 3 | 30.0752 | 30.0847 | -0.0095 |

| | | | | | |
|---|---|---|---|---|---|
| -2 | 6 | 2 | 31.1357 | 31.1023 | 0.0334 |
| -3 | 4 | 0 | 32.5596 | 32.5682 | -0.0086 |
| -1 | 5 | 3 | 33.3631 | 33.3265 | 0.0366 |
| -4 | 0 | 1 | 33.8129 | 33.7995 | 0.0134 |
| -2 | 2 | 4 | 35.4326 | 35.4516 | -0.019 |
| -1 | 0 | 4 | 36.0592 | 36.0696 | -0.0104 |
| -3 | 2 | 4 | 36.4646 | 36.4396 | 0.025 |
| -1 | 2 | 4 | 37.0402 | 37.0131 | 0.0271 |
| -1 | 3 | 4 | 38.1712 | 38.1642 | 0.007 |
| -4 | 3 | 0 | 39.8749 | 39.8159 | 0.059 |
| 1 | 9 | 1 | 40.5121 | 40.5481 | -0.036 |
| -1 | 5 | 4 | 41.6241 | 41.6666 | -0.0425 |
| 3 | 2 | 2 | 43.0536 | 43.0735 | -0.0199 |
| -2 | 1 | 5 | 44.1067 | 44.1071 | -0.0004 |
| 1 | 1 | 4 | 45.93 | 45.9769 | -0.0469 |
| -1 | 7 | 4 | 46.5032 | 46.5121 | -0.0089 |
| 0 | 6 | 4 | 47.3209 | 47.3336 | -0.0127 |
| -5 | 5 | 1 | 48.2914 | 48.276 | 0.0154 |
| -4 | 8 | 4 | 51.5845 | 51.6058 | -0.0213 |
| 1 | 12 | 1 | 52.7499 | 52.7392 | 0.0107 |
| 2 | 2 | 4 | 53.6503 | 53.644 | 0.0063 |
| -1 | 13 | 1 | 55.1186 | 55.1176 | 0.001 |
| 4 | 5 | 2 | 55.9705 | 55.9803 | -0.0098 |
| -6 | 6 | 4 | 57.4181 | 57.4315 | -0.0134 |
| -7 | 1 | 3 | 59.017 | 59.0423 | -0.0253 |
| -7 | 2 | 4 | 59.8432 | 59.8874 | -0.0442 |
| -7 | 4 | 2 | 63.0221 | 63.0165 | 0.0056 |
| 5 | 3 | 3 | 71.9132 | 71.9115 | 0.0017 |
| -5 | 0 | 8 | 73.106 | 73.1115 | -0.0055 |
| -8 | 5 | 2 | 74.7806 | 74.7708 | 0.0098 |

Table S6. Equations used in this work to fit the data of adsorption experiments.

| Serial | Equation | Nmae | Description | Ref. |
| --- | --- | --- | --- | --- |
| **1** | $q_e = \frac{q_m}{1 + K_L C_e}$ | Langmuir | $q_e$ (mg.g$^{-1}$) Adsorption capacity, $C_e$ equilibrium concentration, $q_m$ (mg.g$^{-1}$) is the monolayer saturation capacity constant and $K_L$ (L/mg) is the Langmuir constant associated with the free adsorption energy.<br>The favorability of the adsorption process in the Langmuir model is determined by means of the $R_L$ dimensionless factor ($R_L = 1/(1 + k_L \cdot C_0)$) as follows: $R_L = 0$, $0 < R_L < 1$, $R_L = 1$, and $R_L > 1$ indicating irreversible, favorable, linear, and unfavorable adsorption isotherms, respectively. | [1] |
| **2** | $q_e = K_F C_e^{\frac{1}{n}}$ | Freundlich | $K_F$ Freundlich isotherm constants [(mg/g)/(mg/L)$^{1/n}$], and 1/*n* represents the exponent of non-linearity (i.e., C-type, L-type, and S-type isotherms). *n* is the Freundlich constants, and $n < 1$ indicates poor adsorption while $n = 1$–2 and $n = 2$–10 indicate average and good adsorptions, respectively. The values of *n* and $k_f$ are calculated, respectively | [2] |
| **3** | $q_e = q_m \exp(-\beta\varepsilon^2)$<br>$\varepsilon = RT ln\left(1 + \frac{1}{C_e}\right)$<br>$E_{DR} = \sqrt{\frac{1}{2K_{DR}}}$ | Dubinin–Radushkevich | $q_D$ is the maximum monolayer adsorption capacity (mg/g), $B_D$ is the activity coefficient related to the apparent free energy of adsorbate adsorption onto the adsorbent (mol$^2$/kJ$^2$), $\varepsilon_D$ is the Polanyi potential which is related to the equilibrium concentration, and *E* is the mean adsorption energy. | [3] |
| **4** | $q_e = Q_{max} \frac{RT}{b} \ln(K_T C_e)$ | Temkin | $K_T$ is the Temkin isotherm constant or equilibrium binding constant (L/mg) corresponding to the maximum binding energy, and $b_T$ is the Temkin isotherm constant related to the heat of adsorbate adsorption onto the adsorbent due to adsorbent-adsorbate interaction (J/mol), *R* is the gas constant (8.314 J/mol/K), and *T* is the absolute temperature (herein 298 K). | [4] |

| | | | | |
|---|---|---|---|---|
| **5** | $q_e = \frac{KC_e}{1 + JC_e^n}$ | Jossens | The Jossens adsorption isotherm defines the relationship between adsorbate amount ($q_e$, mg $g^{-1}$) and its concentration ($C_e$, mg $L^{-1}$). The maximum capacity ($q_m$, mg $g^{-1}$) reflects the theoretical limit of filled sites. The affinity constant (K, L $mg^{-1}$) shows interaction strength; higher values mean stronger forces. The heterogeneity factor (n) indicates variance in adsorption behavior values near one suggest Langmuir behavior, while others point to heterogeneous adsorption. These parameters are typically estimated via non-linear regression of experimental data. | [5] |
| **6** | $q_t = q_e\left(1 - e^{K_1 t}\right)$ | Pseudo-First-order kinetic | $q_e$ and $q_t$ are the adsorption capacities at equilibrium and time $t$ (mg/g), and $k_1$ is the rate constant ($min^{-1}$), respectively. | [6] |
| **7** | $q_t = \frac{tK_2 q_e^2}{1 + q_e K_2 t}$ | Pseudo-Second-order kinetic | $k_2$ is the pseudo-second order constant (mg/(g.min)) | [7] |
| **8** | $q_t = K_i t^{1/2} + X$ | Intraparticle diffusion | $q_t$ is the adsorption capacity at time $t$ in (mg/g), $k_{int}$ is the intraparticle diffusion rate constant (mg·$g^{-1}$·$min^{-1/2}$), and $C$ is a constant related to the the thickness of the boundary layer (mg/g). | [8] |
| **9** | $q_t = \frac{1}{\beta}\ln(\alpha\beta t + 1)$ | Elovich | The constants α chemical adsorption rate (mg.$g^{-1}$$min^{-1}$), and β Coefficient in relation with extension of covered surface | [9] |
| **10** | ΔG° = ΔH° − TΔS° | Gibbs free energy | ΔG°: Gibbs free energy change; Kd: equilibrium constant; R: gas constant; T: temperature. | [10] |
| **11** | $LnK_d = \frac{\Delta S^{\circ}}{R} - \frac{\Delta H^{\circ}}{RT}$ | Van't Hoff | ΔS°: entropy change; ΔH°: enthalpy change. | [11] |
| **12** | $LnK_d = lnA - (\frac{Ea^0}{R})\frac{1}{T}$ | Arhinus | Ea was the activation energy, A Arhinus constant, R ideal gas constant 8.314 J/mol K, T (K) is the absolute solution temperature | [12] |

Table S7. The parameter of the adsorption isotherm for Cd(II) ions on FAACP hydrogel beads.

| **Isotherm** | **Value of parameters** | | | |
|---|---|---|---|---|
| | **Parameters** | **25 $^{o}$C** | **35 $^{o}$C** | **45 $^{o}$C** |
| Langmuir | $q_{m\ exp}$ (mg/g) | 254.75 | 355.26 | 425.84 |
| | $q_m$ (mg/g) | 256.82 | 359.2 | 428.8 |
| | $K_L$ (L/mg) | 0.042 | 0.04628 | 0.079 |
| | $R_L$ | 0.17 | 0.15 | 0.10 |
| | Reduced Chi-Sqr | 28.83295 | 49.90252 | 108.64149 |
| | Residual Sum of Squares | 461.32728 | 798.44031 | 1738.26384 |
| | R-Square (COD) | 0.99648 | 0.99658 | 0.99468 |
| | Adj. R-Square | 0.99626 | 0.99637 | 0.99435 |
| | $R^2$ | 0.995 | 0.9961 | 0.9938 |
| Freundlich | n | 2.68 | 2.21 | 0.496 |
| | $K_F$ (mg/g) (L/mg)$^{1/n}$ | 41.089 | 46.9 | 67.21 |
| | Reduced Chi-Sqr | 493.17842 | 333.17728 | 280.8034 |
| | Residual Sum of Squares | 7890.85477 | 5330.83652 | 4212.05094 |
| | R-Square (COD) | 0.93972 | 0.97718 | 0.9848 |
| | Adj. R-Square | 0.93595 | 0.97575 | 0.98379 |
| | $R^2$ | 0.936 | 0.9742 | 0.982 |
| Dubinin–Radushkevich | $Q_{DR}$(mg.g$^{-1}$) | 232.04 | 301.62 | 354.2 |
| | $K_{DR}$ (mol²/kJ²) | 2.40711E-5 | 1.38638E-5 | 4.03151E-6 |
| | Ea (kJ/mol) | 30.18 | 31.62 | 33.2 |
| | Reduced Chi-Sqr | 589.4937 | 1525.39951 | 2277.97539 |
| | Residual Sum of Squares | 9431.89916 | 24406.39216 | 36447.60617 |
| | R-Square (COD) | 0.92794 | 0.89552 | 0.88848 |
| | Adj. R-Square | 0.92344 | 0.88899 | 0.88151 |
| | $R^2$ | 0.924 | 0.884 | 0.882 |
| Temkin | $b_T$ (J/mol) | 39.9 | 29.15 | 24.54 |
| | $K_T$ (L/mol) | 0.432 | 0.57 | 1.14 |
| | Reduced Chi-Sqr | 60.66051 | 68.56615 | 305.5601 |

| | | | | |
|---|---|---|---|---|
| | Residual Sum of Squares | 970.56819 | 1097.05834 | 4888.96157 |
| | R-Square (COD) | 0.99259 | 0.9953 | 0.98504 |
| | Adj. R-Square | 0.99212 | 0.99501 | 0.98411 |
| | $R^2$ | 0.9932 | 0.9952 | 0.985 |
| Jossens | K | 10.78 | 23.06 | 58.6 |
| | J | 0.024 | 0.092 | 0.25 |
| | Reduced Chi-Sqr | 19.16068 | 33.25771 | 43.26554 |
| | Residual Sum of Squares | 287.41026 | 498.86567 | 648.98312 |
| | R-Square (COD) | 0.9978 | 0.99786 | 0.99801 |
| | Adj. R-Square | 0.99751 | 0.99758 | 0.99775 |
| | $R^2$ | 0.9962 | 0.9965 | 0.998 |

Table S8. Models of adsorption kinetic parameters of Cd(II) ions on FAACP hydrogel beads.

| **Model** | **Value of parameters** | | | |
|---|---|---|---|---|
| | **Parameters** | **25 $^oC$** | **35 $^oC$** | **45 $^oC$** |
| Pseudo-First-order kinetic | $K_1$ (min$^{-1}$)x10$^{-2}$ | 0.022 | 0.028 | 0.032 |
| | Reduced Chi-Sqr | 7.90845 | 15.83493 | 22.00265 |
| | Residual Sum of Squares | 150.26052 | 300.86364 | 418.05031 |
| | R-Square (COD) | 0.99865 | 0.99724 | 0.98724 |
| | Adj. R-Square | 0.99858 | 0.9977 | 0.9776 |
| | $R^2$ | 0.996 | 0.9958 | 0.981 |
| Pseudo-second-order kinetic | $K_2$ (g.mg$^{-1}$min$^{-1}$)x10$^{-2}$ | 8.34168E-5 | 5.89495E-5 | 5.00099E-5 |
| | $q_e$ (mg/g) | 255.6 | 358.2 | 422.4 |
| | Reduced Chi-Sqr | 10.03849 | 20.09988 | 27.9288 |
| | Residual Sum of Squares | 190.73137 | 381.89769 | 530.64719 |
| | R-Square (COD) | 0.99829 | 0.9974 | 0.98754 |
| | Adj. R-Square | 0.9982 | 0.9968 | 0.9868 |
| | $R^2$ | 0.9981 | 0.996 | 0.971 |

| | | | | |
|---|---|---|---|---|
| Intraparticle diffusion | $K_i$ (mgg$^{-1}$min$^{1/2}$) | 27.38029 | 38.74 | 45.66 |
| | X (mg/g) | 2.005 | 2.83 | 3.34 |
| | Reduced Chi-Sqr | 155.19909 | 310.75217 | 431.79043 |
| | Residual Sum of Squares | 2948.78262 | 5904.29121 | 8204.01825 |
| | R-Square (COD) | 0.97352 | 0.9728 | 0.97418 |
| | Adj. R-Square | 0.97212 | 0.9734 | 0.97402 |
| | $R^2$ | 0.973 | 0.982 | 0.974 |
| Elovich | β (g/mg) | 77.89 | 110.22 | 129.93 |
| | α (mgg$^{-1}$.min$^{-1}$) | 0.00358 | 0.00253 | 0.00215 |
| | Reduced Chi-Sqr | 26.32628 | 52.71261 | 73.24422 |
| | Residual Sum of Squares | 500.19939 | 1001.53956 | 1391.64017 |
| | R-Square (COD) | 0.99551 | 0.9962 | 0.9881 |
| | Adj. R-Square | 0.99527 | 0.9968 | 0.9876 |
| | $R^2$ | 0.9948 | 0.997 | 0.9854 |
| Experimental data | $q_e$ (exp) (mmol/g) | 253.2 | 361.6 | 425.1 |

Table S9. The thermodynamic parameters.

| T (K) | ΔG (kJ/mol) | ΔH° (kJ/mol) | ΔS° (J/mol.K) |
|---|---|---|---|
| 293 | -0.67826 | | |
| 298 | -2.21302 | 89.25 | 306.95 |
| 303 | -3.74778 | | |
| 308 | -5.28255 | | |
| 313 | -6.81731 | | |
| 318 | -8.35208 | | |

Table S10. Characteristic of the real water samples.

| **Parameter** | **Tap Water** | **Sea Water** | **Waste water** |
|---|---|---|---|
| pH | 7.6 | 8.4 | 4.8 |
| Total Dissolved Solids (mg/L) | 384.8 | 22177 | 3564 |
| Turbidity (NTU) | 1.4 | 3.1 | 30.64 |
| Electrical Conductivity (μS/cm) | 580 | 33600 | 5400 |
| Total Hardness (as $CaCO_3$, mg/L) | 194 | 5850 | 740 |
| $Mg^{2+}$ (mg/L) | 26 | 1280 | 136 |
| $Ca^{2+}$ (mg/L) | 74 | 396 | 184 |
| $K^{+}$ (mg/L) | 6.1 | 374 | 62 |
| $Cl^{-}$ (mg/L) | 114 | 20600 | 1360 |
| $Na^{+}$ (mg/L) | 72 | 9450 | 870 |
| $HCO_3^{-}$ (mg/L) | 186 | 131 | 320 |
| $SO_4^{2-}$ (mg/L) | 84 | 3250 | 614 |
| Fe (mg/L) | 0.06 | 0.61 | 3.2 |
| Mn (mg/L) | 0.05 | 0.08 | 1.4 |
| Ni (mg/L) | 0.60 | 0.70 | 2.8 |
| Zn (mg/L) | 0.16 | 0.12 | 12.4 |
| Chemical Oxygen Demand (COD, mg/L) | 20 | 36 | 845 |
| Total Organic Carbon (TOC, mg/L) | 2.84 | 6.2 | 188 |
| Residual Chlorine (mg/L) | 0.56 | 0.4 | ND |
| Dominant Interfering Substances | $Ca^{2+}$, $Mg^{2+}$, $HCO_3^{-}$ | $Na^{+}$, $Cl^{-}$, $SO_4^{2-}$ | $Zn^{2+}$, $Fe^{2+}$, $Cu^{2+}$, organics |
| Cd(II) Removal Efficiency (%) | 96.2 | 82.6 | 91.2 |

Table S11. Comparison of Cd(II) ions adsorption capacity in various adsorbents.

| Materials | $Q_e$ (mg/g) | Initial concentration (mg/L) | pH | Adsorbent dose (g/L) | Ref. |
|---|---|---|---|---|---|
| *Canna indica*-derived biochar | 188.8 | 10–300 | 5 | 0.5 | [13] |
| Methylisothiocyanate decorated PAMAM dendrimer/mesoporous silica | 97.8 | 10–200 | 6 | 0.2 | [14] |
| Cellulose/ ethylenediaminetetraacetic acid | 33.2 | 10–150 | 5.5 | 1 | [15] |
| Carboxymethyl cellulose/polyacrylamide | 256.4 | 20–300 | 6 | 0.5 | [16] |
| $TiO_2$/glutaraldehyde/carboxymethyl cellulose | 274.28 | 10–300 | 5.5 | 0.5 | [17] |
| Graphene oxide/carboxymethyl cellulose | 46.13 | 10–200 | 5 | 0.5 | [18] |
| Poly(vinyl alcohol)/chitosan | 148 | 10–200 | 5.5 | 0.5 | [19] |
| Poly(glycidyl methacrylate)/cellulose/ iminodiacetic acid | 53.4 | 10–150 | 6 | 0.3 | [20] |
| Chelating polyacrylonitrile | 146 | 10–200 | 5.5 | 0.5 | [21] |
| Polyacrylonitrile/Na-Y-zeolite/amidoxime | 39.4 | 10–150 | 5 | 0.5 | [22] |
| Cellulose nanocrystalline | 57.76 | 10–200 | 5.5 | 0.5 | [23] |
| Amidoxime chelating resin | 20.7 | 10–100 | 5 | 0.5 | [24] |
| Blue algae-derived biochar | 135.7 | 10–250 | 5.5 | 0.5 | [25] |
| Fe/Zn/*durian shells* biochar | 99.83 | 10–200 | 5 | 0.5 | [26] |
| Struvite/attapulgite | 121.14 | 10–250 | 6 | 0.5 | [27] |
| Biochar derived-sewage | 127.9 | 20–300 | 5.5 | 0.5 | [28] |

| | | | | | |
|---|---|---|---|---|---|
| sludge/calcium sulfate | | | | | |
| Danthron/MWCNTs | 52.9 | 10–200 | 5.5 | 0.2 | [29] |
| Hydroxyapatite | 12.36 | 10–100 | 6 | 1 | [30] |
| Ca-Mg phosphate based on dolomite | 241.7 | 20–300 | 5.5 | 0.5 | [31] |
| Unmodifed Cel | 13.6 | 10–100 | 5 | 0.5 | [32] |
| Cel/PAN/AO | 123.23 | 10–200 | 5.5 | 0.5 | [32] |
| FAACP Hydrogel beads | 254.57 | 10–300 | 6 | 0.8 | This wor |

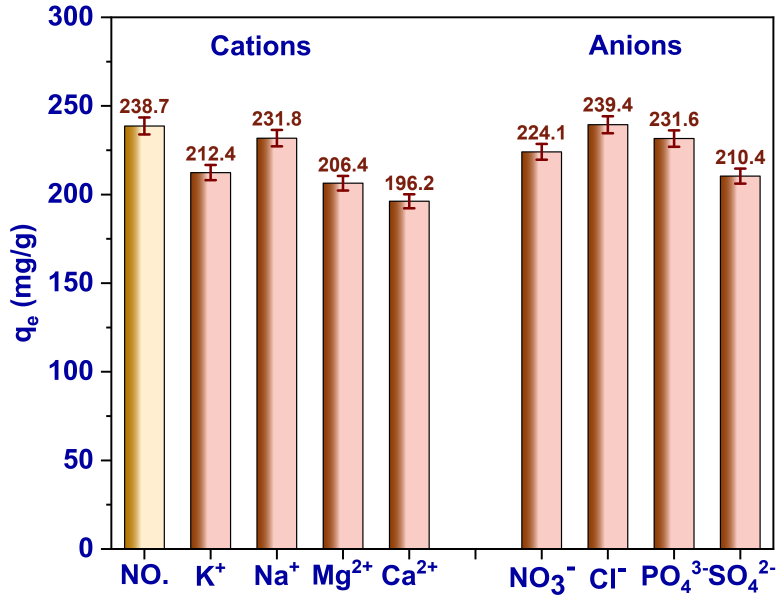


Fig. S1. Effect of interference ions.

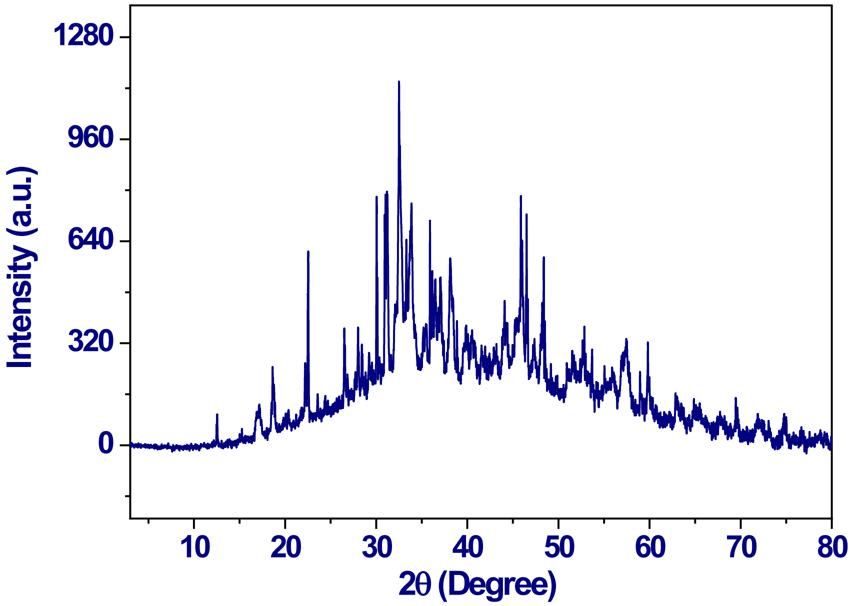


Fig. S2. XRD pattern of regenerated FAACP.

# References


[1] I. Langmuir, The constitution and fundamental properties of solids and liquids. Part I. Solids, J. Am. Chem. Soc., 38 (1916) 2221-2295.

[2] H.M.F. Freundlich, Over the adsorption in solution, J. Phys. Chem., 57 (1906) 385-471.

[3] M. Dubinin, The equation of the characteristic curve of activated charcoal, Proc. Acad. Sci. USSR Phys. Chem. Sect., 55 (1947) 327-329.

[4] V.P. M.I. Tempkin, Kinetics of ammonia synthesis on promoted iron catalyst, Acta Phys. Chim. USSR, 12 (1940) 327-356.

[5] J. Bayuo, K.B. Pelig-Ba, M.A. Abukari, Isotherm modeling of lead (II) adsorption from aqueous solution using groundnut shell as a low-cost adsorbent, IOSR J Appl Chem (IOSR-JAC), 11 (2018) 18-23.

[6] S.K. Lagergren, About the theory of so-called adsorption of soluble substances, Sven. Vetenskapsakad. Handingarl, 24 (1898) 1-39.

[7] Y.-S. Ho, G. McKay, Sorption of dye from aqueous solution by peat, Chemical engineering journal, 70 (1998) 115-124.

[8] W.J. Weber Jr, J.C. Morris, Kinetics of adsorption on carbon from solution, J. Sanit. Eng. Div., 89 (1963) 31-59.

[9] M.H. Dehghani, A. Dehghan, A. Najafpoor, Removing Reactive Red 120 and 196 using chitosan/zeolite composite from aqueous solutions: Kinetics, isotherms, and process optimization, Journal of Industrial and Engineering Chemistry, 51 (2017) 185-195.
[10] E.C. Lima, A. Hosseini-Bandegharaei, J.C. Moreno-Piraján, I. Anastopoulos, A critical review of the estimation of the thermodynamic parameters on adsorption equilibria. Wrong use of equilibrium constant in the Van't Hoof equation for calculation of thermodynamic parameters of adsorption, Journal of molecular liquids, 273 (2019) 425-434.
[11] H.N. Tran, S.-J. You, A. Hosseini-Bandegharaei, H.-P. Chao, Mistakes and inconsistencies regarding adsorption of contaminants from aqueous solutions: a critical review, Water research, 120 (2017) 88-116.
[12] B. Oladipo, E. Govender-Opitz, T.V. Ojumu, Kinetics, thermodynamics, and mechanism of Cu (II) ion sorption by biogenic iron precipitate: using the lens of wastewater treatment to diagnose a typical biohydrometallurgical problem, ACS omega, 6 (2021) 27984-27993.
[13] X. Cui, S. Fang, Y. Yao, T. Li, Q. Ni, X. Yang, Z. He, Potential mechanisms of cadmium removal from aqueous solution by Canna indica derived biochar, Science of the Total Environment, 562 (2016) 517-525.
[14] L. Luan, B. Tang, Y. Liu, W. Xu, Y. Liu, A. Wang, Y. Niu, Direct synthesis of sulfur-decorating PAMAM dendrimer/mesoporous silica for enhanced Hg (II) and Cd (II) adsorption, Langmuir, 38 (2022) 698-710.
[15] A. Daochalermwong, N. Chanka, K. Songsrirote, P. Dittanet, C. Niamnuy, A. Seubsai, Removal of heavy metal ions using modified celluloses prepared from pineapple leaf fiber, ACS omega, 5 (2020) 5285-5296.
[16] C.B. Godiya, X. Cheng, D. Li, Z. Chen, X. Lu, Carboxymethyl cellulose/polyacrylamide composite hydrogel for cascaded treatment/reuse of heavy metal ions in wastewater, Journal of hazardous materials, 364 (2019) 28-38.
[17] M.E. Mahmoud, A.E. Abdou, M.E. Sobhy, N.A. Fekry, Solid–solid crosslinking of carboxymethyl cellulose nanolayer on titanium oxide nanoparticles as a novel biocomposite for efficient removal of toxic heavy metals from water, International journal of biological macromolecules, 105 (2017) 1269-1278.
[18] Y. Zhang, Y. Liu, X. Wang, Z. Sun, J. Ma, T. Wu, F. Xing, J. Gao, Porous graphene oxide/carboxymethyl cellulose monoliths, with high metal ion adsorption, Carbohydrate polymers, 101 (2014) 392-400.
[19] M.R. Karim, M.O. Aijaz, N.H. Alharth, H.F. Alharbi, F.S. Al-Mubaddel, M.R. Awual, Composite nanofibers membranes of poly (vinyl alcohol)/chitosan for selective lead (II) and cadmium (II) ions removal from wastewater, Ecotoxicology and environmental safety, 169 (2019) 479-486.
[20] M. Barsbay, P.A. Kavaklı, S. Tilki, C. Kavaklı, O. Güven, Porous cellulosic adsorbent for the removal of Cd (II), Pb (II) and Cu (II) ions from aqueous media, Radiation Physics and Chemistry, 142 (2018) 70-76.
[21] P. Bhunia, S. Chatterjee, P. Rudra, S. De, Chelating polyacrylonitrile beads for removal of lead and cadmium from wastewater, Separation and Purification Technology, 193 (2018) 202-213.
[22] K. Elwakeel, A. El-Bindary, E. Kouta, E. Guibal, Functionalization of polyacrylonitrile/Na-Y-zeolite composite with amidoxime groups for the sorption of Cu (II), Cd (II) and Pb (II) metal ions, Chemical Engineering Journal, 332 (2018) 727-736.
[23] H.T. Kara, S.T. Anshebo, F.K. Sabir, Adsorptive removal of Cd (II) ions from wastewater using maleic anhydride nanocellulose, Journal of Nanotechnology, 2021 (2021) 9966811.
[24] C. Zheng, C. He, Y. Yang, T. Fujita, G. Wang, W. Yang, Characterization of waste amidoxime chelating resin and its reutilization performance in adsorption of Pb (II), Cu (II), Cd (II) and Zn (II) Ions, Metals, 12 (2022) 149.
[25] P. Liu, D. Rao, L. Zou, Y. Teng, H. Yu, Capacity and potential mechanisms of Cd (II) adsorption from aqueous solution by blue algae-derived biochars, Science of the Total Environment, 767 (2021) 145447.

[26] T. Yang, Y. Xu, Q. Huang, Y. Sun, X. Liang, L. Wang, X. Qin, L. Zhao, Adsorption characteristics and the removal mechanism of two novel Fe-Zn composite modified biochar for Cd (II) in water, Bioresource technology, 333 (2021) 125078.
[27] H. Wang, X. Wang, J. Ma, P. Xia, J. Zhao, Removal of cadmium (II) from aqueous solution: a comparative study of raw attapulgite clay and a reusable waste–struvite/attapulgite obtained from nutrient-rich wastewater, Journal of hazardous materials, 329 (2017) 66-76.
[28] L. Liu, T. Yue, R. Liu, H. Lin, D. Wang, B. Li, Efficient absorptive removal of Cd ( Ⅱ ) in aqueous solution by biochar derived from sewage sludge and calcium sulfate, Bioresource Technology, 336 (2021) 125333.
[29] T. Shahryari, P. Singh, P. Raizada, A. Davidyants, L. Thangavelu, S. Sivamani, A. Naseri, F. Vahidipour, A. Ivanets, A. Hosseini-Bandegharaei, Adsorption properties of Danthron-impregnated carbon nanotubes and their usage for solid phase extraction of heavy metal ions, Colloids and surfaces A: physicochemical and engineering aspects, 641 (2022) 128528.
[30] A. Ivanets, N. Kitikova, I. Shashkova, M.Y. Roshchina, V. Srivastava, M. Sillanpää, Adsorption performance of hydroxyapatite with different crystalline and porous structure towards metal ions in multicomponent solution, Journal of Water Process Engineering, 32 (2019) 100963.
[31] A. Ivanets, V. Srivastava, N. Kitikova, I. Shashkova, M. Sillanpää, Non-apatite Ca-Mg phosphate sorbent for removal of toxic metal ions from aqueous solutions, Journal of environmental chemical engineering, 5 (2017) 2010-2017.
[32] H.A. Abdelmonem, T.F. Hassanein, H.E. Sharafeldin, H. Gomaa, A.S. Ahmed, A.M. Abdel-lateef, E.M. Allam, M.F. Cheira, M.E. Eissa, A.H. Tilp, Cellulose-embedded polyacrylonitrile/amidoxime for the removal of cadmium (II) from wastewater: Adsorption performance and proposed mechanism, Colloids and Surfaces A: Physicochemical and Engineering Aspects, 684 (2024) 133081.
